# A Heterodimeric Reduced-Ferredoxin-Dependent Methylenetetrahydrofolate Reductase from Syngas-Fermenting Clostridium ljungdahlii

**DOI:** 10.1128/Spectrum.00958-21

**Published:** 2021-10-13

**Authors:** Jihong Yi, Haiyan Huang, Jiyu Liang, Rufei Wang, Ziyong Liu, Fuli Li, Shuning Wang

**Affiliations:** a State Key Laboratory of Microbial Technology, Microbial Technology Institute, Shandong University, Qingdao, People’s Republic of China; b School of Basic Medicine, Shandong First Medical University & Shandong Academy of Medical Science s, Jinan, People’s Republic of China; c Qingdao Institute of Bioenergy and Bioprocess Technology, Chinese Academy of Sciences, Qingdao, People’s Republic of China; University of Southern Denmark

**Keywords:** methylenetetrahydrofolate reductase, ferredoxin, Wood-Ljungdahl pathway, energy metabolism, *Clostridium ljungdahlii*

## Abstract

The strict anaerobe Clostridium ljungdahlii can ferment CO or H_2_/CO_2_ via the Wood-Ljungdahl pathway to acetate, ethanol, and 2,3‐butanediol. This ability has attracted considerable interest, since it can be used for syngas fermentation to produce biofuels and biochemicals. However, the key enzyme methylenetetrahydrofolate reductase (MTHFR) in the Wood-Ljungdahl pathway of the strain has not been characterized, and its physiological electron donor is unclear. In this study, we purified the enzyme 46-fold with a benzyl viologen reduction activity of 41.2 U/mg from C. ljungdahlii cells grown on CO. It is composed of two subunits, MetF (31.5 kDa) and MetV (23.5 kDa), and has an apparent molecular mass of 62.2 kDa. The brownish yellow protein contains 0.73 flavin mononucleotide (FMN) and 7.4 Fe, in agreement with the prediction that MetF binds one flavin and MetV binds two [4Fe4S] clusters. It cannot use NAD(P)H as its electron donor or catalyze an electron-bifurcating reaction in combination with ferredoxin as an electron acceptor. The reduced recombinant ferredoxin, flavodoxin, and thioredoxin of *C. ljungdahlii* can serve as electron donors with specific activities of 91.2, 22.1, and 7.4 U/mg, respectively. The apparent *K_m_* values for reduced ferredoxin and flavodoxin were around 1.46 μM and 0.73 μM, respectively. Subunit composition and phylogenetic analysis showed that the enzyme from *C. ljungdahlii* belongs to MetFV-type MTHFR, which is a heterodimer, and uses reduced ferredoxin as its electron donor. Based on these results, we discuss the energy metabolism of *C. ljungdahlii* when it grows on CO or H_2_ plus CO_2_.

**IMPORTANCE** Syngas, a mixture of CO, CO_2_, and H_2_, is the main component of steel mill waste gas and also can be generated by the gasification of biomass and urban domestic waste. Its fermentation to biofuels and biocommodities has attracted attention due to the economic and environmental benefits of this process. Clostridium ljungdahlii is one of the superior acetogens used in the technology. However, the biochemical mechanism of its gas fermentation via the Wood-Ljungdahl pathway is not completely clear. In this study, the key enzyme, methylenetetrahydrofolate reductase (MTHFR), was characterized and found to be a non-electron-bifurcating heterodimer with reduced ferredoxin as its electron donor, representing another example of MetFV-type MTHFR. The findings will form the basis for a deeper understanding of the energy metabolism of syngas fermentation by *C. ljungdahlii*, which is valuable for developing metabolic engineering strains and efficient syngas fermentation technologies.

## INTRODUCTION

Acetogenic bacteria are strict anaerobes which can fix CO_2_ through the Wood-Ljungdahl pathway (WLP) (1, 2). They can grow autotrophically using syngas, a mixture of CO, CO_2_, and H_2_, as their source of carbon and energy and produce biofuels and chemicals such as acetate, ethanol, butanol, and 2,3-butanediol (3). This process can not only capture CO_2_ to reduce its emission but also make wastes recyclable through directly fermenting the waste gases from steel factories or those from waste gasification. The use of acetogens in the process has attracted considerable attention as the basis for a new and valuable biomanufacturing platform (4).

The WLP is thought to be the most energetically efficient pathway by converting two molecules of CO_2_ to one molecule of acetate (5, 6). It consists of two separate branches. In the methyl branch, a molecule of CO_2_ is first reduced to formate, which then combines with one-carbon-carrier tetrahydrofolate (THF) to produce formyl-THF at the cost of a molecule of ATP. Subsequently, formyl-THF is cyclodehydrated to generate methenyl-THF, which is then reduced to methylene-THF and methyl-THF. Finally, methyl-THF is condensed with CO formed by the reduction of the second CO_2_ molecule via the carbonyl branch to produce acetyl-CoA, releasing THF. Acetyl coenzyme A (acetyl-CoA) is further transformed into acetate by the catalysis of phosphotransacetylase (Pta) and acetate kinase (Ack), in which a molecule of ATP is yielded by substrate-level phosphorylation. Thus, the net ATP gain is zero in the process of fixing CO_2_ to generate acetate (7, 8).

Considerable research has been carried out into the coupling of WLP to ATP synthesis. The main theory to date has been that in the WLP, ferredoxin (Fd) is reduced by CO or H_2_ and the reduced Fd (Fd_red_^2−^) is further used to reduce NAD^+^ by membrane-associated ferredoxin:NAD^+^ oxidoreductase (Rnf) or H^+^ by the energy-converting hydrogenase (Ech) (9, 10). This reaction is accompanied by “pumping” of the protons or sodium ions out of the membrane, forming a transmembrane ion gradient, which is coupled with the F_1_F_0_ATPase-catalyzed reaction on the membrane to promote the synthesis of ATP. Furthermore, an energy-coupling mechanism called flavin-based electron bifurcation plays a key role in the energy conservation. In this mechanism, an endergonic reaction is coupled to an exergonic reaction catalyzed by a group of flavin-containing oxidoreductases and Fd is required as one of substrates (11–13).

*C. ljungdahlii* is one of the most promising strains for fermenting syngas to produce biofuels and biocommodities (14). Methylenetetrahydrofolate reductase (MTHFR), a key enzyme in the WLP, has attracted considerable attention (15–17), because in the WLP, the methylene-THF/methyl-THF couple has the highest redox potential (E_0_′ = −200 mV) and is considered to be an important energy-conserving site (18, 19). In some acetogens, the MTHFRs have been characterized. In Blautia producta, MTHFR has only one subunit MetF, as does the enzyme from aerobes such as Escherichia coli (20, 21). The MTHFR from Acetobacterium woodii was found to be a trimer composed of MetF, MetV, and RnfC2 subunits (22), while in Moorella thermoacetica it is a hexamer containing additional HdrABC and MvhD subunits other than MetFV (23). All these MTHFRs have been reported to use NADH as their physiological electron donors, although the MTHFR from M. thermoacetica is suggested to also be an electron-bifurcating enzyme. Differently, Clostridium formicoaceticum MTHFR consists of only MetFV and can use Fd_red_^2−^ or FADH_2_ as its electron donor (24). Similarly, MTHFR enriched from Clostridium aceticum was found to catalyze the methylene-THF-dependent oxidation of methyl viologen (MV) with an activity of around 400 U/mg (25). In a recent study, MTHFR from Eubacterium callanderi KIST612 was shown to be composed of MetFV, and both methylene-THF-dependent oxidation of MV activity (644 U/mg) and Fd-dependent methylene-THF reduction activity (1.5 U/mg) were measured (26). Genomic analysis of MTHFR in C. ljungdahlii/Clostridium autoethanogenum showed that it also contains only the MetFV genes but not those for RnfC2 or HdrABCMvhD. The characterization of this enzyme is urgently needed, since it is fundamental to elucidate the energy metabolism in this acetogen.

In recent years, two hypotheses about the MTHFR from *C. ljungdahlii*/C. autoethanogenum have been proposed. One is that it is an NADH- and Fd-dependent electron-bifurcating enzyme, and the other is that it is NADH-dependent but non-electron-bifurcating (16, 17, 19, 27). However, the binding site of NAD(P)H was not detected on MetFV in all the multimeric MTHFRs from acetogens, while it was found on the RnfC subunit of the enzyme from A. woodii and the HdrA subunit of M. thermoacetica (22, 23). Therefore, if *C. ljungdahlii* MTHFR uses NAD(P)H as its electron donor, it might contain another subunit for binding NAD(P)H, which may bind tightly to MetFV or just interact with it during the reaction. In the genome of *C. ljungdahlii*, there is an *lpd*A gene annotated as encoding dihydrolipoamide dehydrogenase (DLDH) and occurring adjacent to the *metFV* genes in the WLP gene cluster. The predicted DLDH has a binding site for NADH, and its reaction product, dihydrolipoamide, is used as an electron donor for some reductases, such as pyruvate dehydrogenase multienzyme complexes and glycine dehydrogenase (28, 29). We therefore hypothesized that there may be a coupling reaction between MTHFR and DLDH in *C. ljungdahlii.* In addition, redox proteins such as Fd, flavodoxin (Fld), and thioredoxin (Trx) are often used as electron donors for reductases in anaerobes (30), and they all have a low redox potential sufficient to reduce methylene-THF, suggesting that they may function as the electron donor for MTHFR.

In this work, we purified the MTHFR from *C. ljungdahlii* and characterized its enzymatic properties. The possible electron donors, such as NAD(P)H, DLDH, Fd, Fld, and Trx, and the electron-bifurcating reaction were tested, and the electron transfer proteins used are all from *C. ljungdahlii*. The results indicate that *C. ljungdahlii* MTHFR uses Fd_red_^2−^ as its electron donor, and electron bifurcation is not involved in the reaction. Based on these findings, we discuss the energy metabolism of *C. ljungdahlii* growing on CO or H_2_ plus CO_2_. By coincidence, MTHFR from *C. ljungdahlii* was also reported by Volker Müller’s group in a very recent study (31) when the manuscript was submitted. Although most results presented here are in agreement with them, we reported a higher enzyme activity determined using a different detection method, tested the activity with various intrinsic electron donors in *C. ljungdahlii*, advanced the classification and distribution of MTHFR, and discussed the energy metabolism of *C. ljungdahlii* from different views.

## RESULTS

### Purification of MTHFR from *C. ljungdahlii* cells grown on CO.

The enzyme was purified from *C. ljungdahlii* cells under strict anoxic conditions by following the benzyl viologen (BV) reduction activity with methyl-THF. The activity in the cell extracts was measured to be around 0.9 U/mg. Using a four-step purification procedure (Table 1), the enzyme, which has a brownish yellow color, was successfully purified 46-fold, and the specific activity of BV reduction was 41.2 U/mg. The enzyme lost its activity quickly when exposed to O_2_.

**TABLE 1:** Purification of the MTHFR from *C. ljungdahlii* cells grown on CO

Purification step	Protein (mg)	MTHFR activity (U/mg)^*a*^	Yield (%)	Purification (fold)
Cell extracts	673.1	0.9	100.0	1.0
Ammonium sulfate precipitation	201.9	2.4	80.0	2.7
Phenyl-Sepharose	17.2	18.3	51.9	20.3
Q-Sepharose	5.0	38.3	31.6	42.6
DEAE-Sepharose	4.5	41.2	30.6	45.8

a The MTHFR activity was determined by monitoring methyl-THF-dependent reduction of BV at 555 nm.

**FIG 1:**
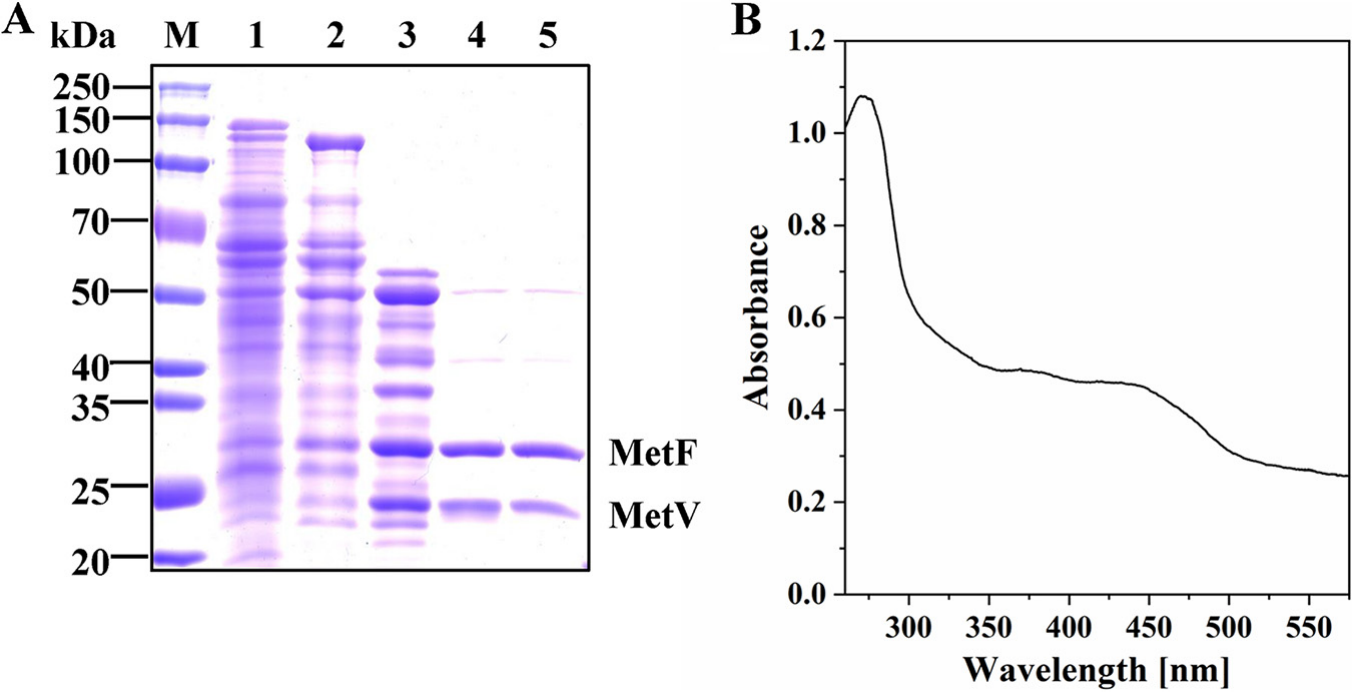
SDS-PAGE analysis (A) and UV-visible absorption spectrum (B) of the purified MTHFR from *C. ljungdahlii*. (A) M, protein marker; lane 1, cell extracts; lane 2, pooled fractions from ammonium sulfate precipitation; lane 3, pooled fractions from phenyl-Sepharose; lane 4, pooled fractions from Q-Sepharose; lane 5, pooled fractions from DEAE-Sepharose. The impurities in lanes 4 and 5 were determined by peptide mass fingerprinting analysis to be dihydrolipoamide dehydrogenase (DLDH, around 50 kDa) and predicted aminotransferase (around 40 kDa). (B) The spectrum of the enzyme was recorded in 50 mM Tris-HCl (pH 7.4) containing 2 mM DTT.

Sodium dodecyl sulfate polyacrylamide gel electrophoresis (SDS-PAGE) analysis showed two visible protein bands (Fig. 1A), which were consistent with the masses deduced from the protein sequences of MetF (31.5 kDa) and MetV (23.5 kDa). Densitometry of the SDS-PAGE gel showed a stoichiometry of around 1:1. Peptide mass fingerprinting analysis further confirmed that the enzyme is composed of two subunits (Fig. S1): MetF (locus tag in GenBank: CLJU_RS18520) and MetV (locus tag in GenBank: CLJU_RS18525). Gel filtration showed two close peaks (Fig. S2A and B): the small one corresponds to an apparent molecular mass of 132.3 kDa, and the big one corresponds to an apparent molecular mass of 62.2 kDa, suggesting that the enzyme has both heterodimeric and tetrameric forms and is mainly in a dimeric form. Native PAGE and activity staining (Fig. S2C) showed that the enzyme appeared as a single band with the same mobility during the purification and indicated that the enzyme complex was active and did not disassociate in the process of the purification.

In the purification step of ion-exchange chromatography by the Q-Sepharose column, a yellow protein was eluted at 0.1 M NaCl, just before MTHFR came out (eluted at 0.18 M NaCl). SDS-PAGE analysis showed that it was a single band with an apparent molecular mass around 50 kDa (Fig. S2D). Peptide mass fingerprinting analysis indicated that it was DLDH (encoded by *lpd*A). The enzyme presented a specific activity of 9.2 U/mg for NADH-dependent reduction of lipoamide.

### Cofactor content of the MTHFR of *C. ljungdahlii*.

The UV-visible absorption spectrum showed that the purified MTHFR of *C. ljungdahlii* had the characteristic peak of flavin (380 nm and 450 nm) (Fig. 1B), and high-performance liquid chromatography (HPLC) analysis further confirmed that the MTHFR contains flavin mononucleotide (FMN) as the cofactor. The purified protein was determined to contain 0.73 FMN and 7.4 Fe, which is in agreement with the prediction from its protein sequence that it binds one flavin on MetF and two [4Fe4S] clusters on MetV. In bacteria, except for a few MTHFRs without a flavin (32), most MTHFRs composed of only MetF subunits bind a flavin adenine dinucleotide (FAD) rather than FMN (20, 21, 33–35). In contrast, the MTHFRs of *A. woodii* (trimeric complex) (22) and *M. thermoacetica* (hexameric complex) (23) bind an FMN rather than FAD on their MetF subunit, similar to the MTHFR from *C. ljungdahli*. The MTHFR from C. formicoaceticum is exceptional and binds an FAD instead of FMN (24). The MetV of *C. ljungdahlii*, like *A. woodii*, contains two [4Fe4S] clusters, while the MetV subunit of *C. formicoaceticum* and *M. thermoacetica* contains one [4Fe4S] cluster.

### Heterologous expression and purification of the recombinant MetFV and DLDH of *C. ljungdahlii*.

MetFV and DLDH were expressed in E. coli C41 and purified through a Ni-Sepharose column. The recombinant MetFV had a yield of 30 mg protein per liter culture and presented two bands with apparent molecular masses of 33 kDa and 24 kDa, as revealed by SDS-PAGE analysis (Fig. S3A). The purified protein was brownish and showed the characteristic peaks (380 nm and 450 nm) for flavins in its UV-visible absorption spectrum (Fig. S3B). These features are identical to those of the wild-type MetFV purified from *C. ljungdahlii* cells. However, the recombinant MTHFR had a low activity for BV reduction (1.8 U/mg), indicating that it was not well expressed, as revealed by the low iron content (4.5 mol Fe per mol protein). We also tried to make constructs for encoding a His tag at different termini of the enzyme complex or without any tag, express it in other hosts such as E. coli BL21(DE3)-groELS, add riboflavin and ZnCl_2_ to the medium, induce it under anaerobic conditions, and reconstitute FeS clusters *in vitro* (36). However, the enzyme activity was not improved (data not shown). The results might be caused by incorrect folding and iron-sulfur cluster assembly during the heterologous expression.

**FIG 2:**
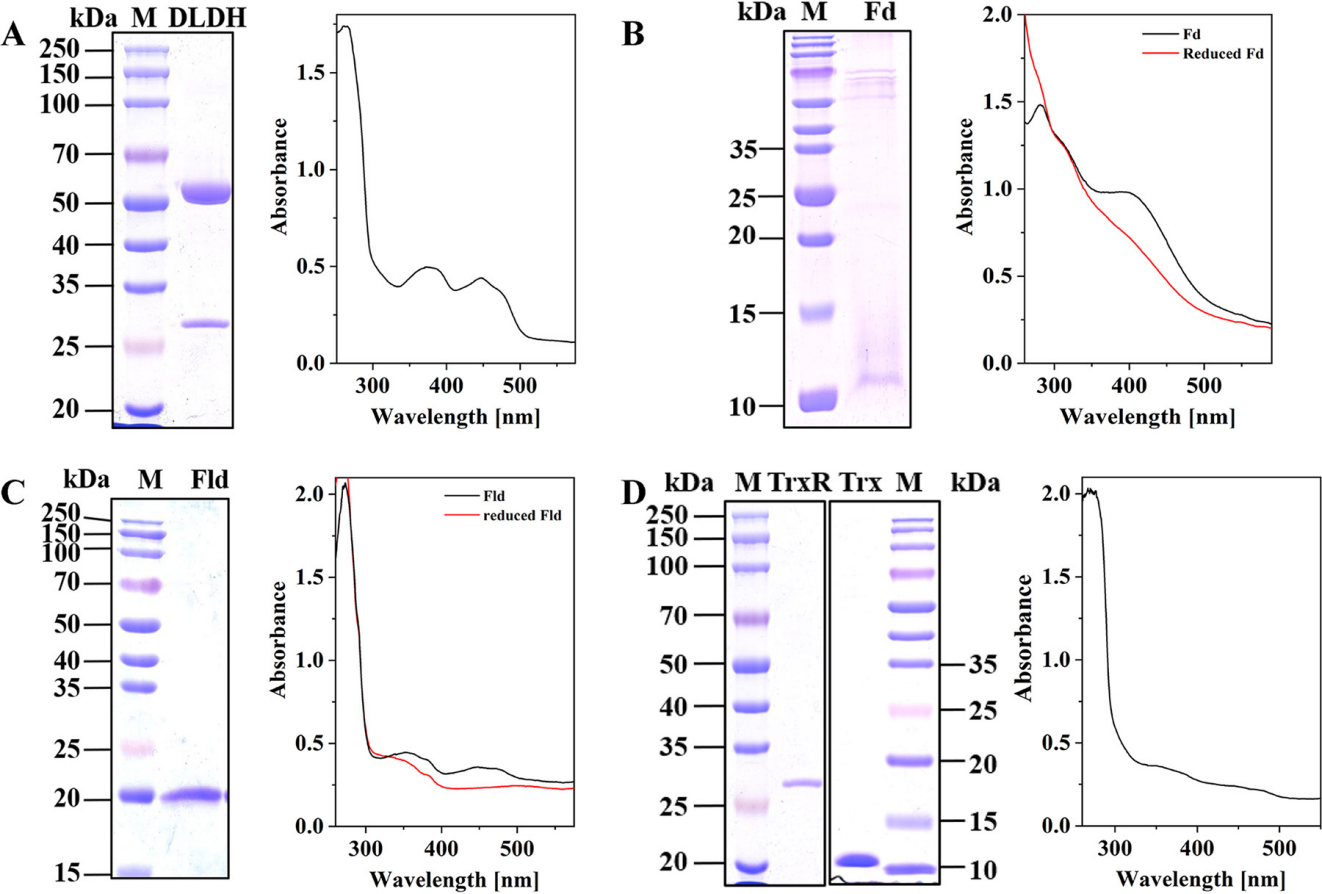
SDS-PAGE analyses and UV-visible absorption spectra of the purified recombinant redox proteins of *C. ljungdahlii*. (A) DLDH, the spectrum shows the characteristic peaks of flavin (380 and 450 nm). (B) Fd, the His tag is on the C terminus. The spectrum of Fd shows the characteristic peak of iron-sulfur cluster (390 nm). After the Fd was reduced with sodium dithionite, the absorption peak for iron-sulfur cluster disappeared. (C) Fld, the spectrum shows the characteristic peaks of flavin (380 and 450 nm). After reducing with sodium dithionite, the absorption peak for flavin disappeared. (D) TrxR and Trx, the spectrum of TrxR shows the characteristic peaks of flavin (380 and 450 nm).

The purified recombinant DLDH had a yellow color and produced a single band with an apparent molecular mass of 51 kDa in SDS-PAGE analysis (Fig. 2A). The UV-visible absorption spectrum showed the characteristic peaks (380 nm and 450 nm) of the flavin. It can catalyze the reduction of lipoamide with NADH with a specific activity of 3.2 U/mg, and neither NADPH nor Fd_red_^2−^ could serve as the electron donor. The preparation was used in subsequent experiments for determining the activity of MTHFR from *C. ljungdahlii*.

### Heterologous expression and purification of the recombinant Fd, Fld, Trx, and TrxR of *C. ljungdahlii*.

The red-brown preparations of recombinant Fd (locus tag in GenBank: CLJU_RS00885) were obtained after purification with a yield of 20 mg per liter culture. SDS-PAGE analysis revealed one band with some dispersion, which indicated a molecular mass of more than 10 kDa, larger than the predicted size of 7 kDa for Fd (Fig. 2B). This might be caused by the properties of the iron-sulfur clusters in the protein, which can bind to the SDS particles in the gel to disperse the protein (37). The UV-visible absorption spectrum showed a characteristic peak of iron-sulfur clusters at 390 nm, which disappeared after reduction with dithionite. An *A*_390nm_/*A*_280nm_ of 0.67 was measured, indicating that the iron-sulfur clusters in most molecules were well synthesized. These properties are similar to the 2[4Fe4S]-type Fd from Clostridium pasteurianum (38).

The recombinant Fld (locus tag in GenBank: CLJU_RS06875) was purified, yielding 12 mg per liter culture. The purified protein was purple in color, because the buffer used for the purification contained 2 mM dithiothreitol (DTT), and Fld was reduced to a semiquinone state under these conditions (39). SDS-PAGE analysis revealed only one band, with a molecular mass of 20 kDa (Fig. 2C), larger than the predicted size of 16 kDa, because it contained a His tag at both the N and C termini of the protein. The UV-visible absorption spectrum showed the characteristic peaks of flavin (380 nm and 450 nm) (Fig. 2C). By HPLC analysis, it was found to contain an FMN, similar to the Flds from other bacteria (39, 40).

The recombinant Trx and TrxR (locus tags in GenBank: CLJU_RS19940-5) were purified, yielding 14 mg and 20 mg protein per liter culture, respectively. TrxR appeared bright yellow, while Trx protein appeared colorless. SDS-PAGE revealed a single band for the TrxR preparation, with an apparent molecular mass of 31 kDa, and one band for the Trx preparation, with an apparent molecular mass of 11 kDa (Fig. 2D). The UV light-visible absorption spectrum of TrxR showed the characteristic peaks of flavins (380 nm and 450 nm) (Fig. 2D). The enzyme activity of the recombinant thioredoxin reduction system was determined, and only NADPH could serve as the electron donor to reduce Trx, with a specific activity of 0.9 U/mg, similar to that of the enzyme from Clostridium litorale, Clostridium sporogenes, and Clostridium cyclindrosporum (41). This reduction system was used in the subsequent experiments.

### NAD(P)H and DLDH could not supply electrons for MTHFR of *C. ljungdahlii*.

The common electron donors NADH and NADPH were used to test the enzyme activity of the purified wild-type MTHFR. The possible electron bifurcation reactions for the reduction of methylene-THF were also determined by supplementing recombinant Fd as the second electron acceptor. However, no activity was detected for any of the tests (Table S1).

The possible coupling reaction between DLDH and MTHFR was also investigated (Table S2). Both wild-type and recombinant DLDHs were tested in the experiments. The reactions are based on the assumption that the electrons are first transferred from NADH to lipoamide and then to methylene-THF or that DLDH functions similarly to RnfC2 and HdrA in MTHFR complexes from *A. woodii* and *M. thermoacetica*, which serves as the NADH-oxidizing electron transfer subunit in the complex. However, the results showed that these assumptions are not true. We also measured the possible electron bifurcation reaction using NADH, Fd, lipoamide, and methylene-THF, and no activity was detected. These results indicate that DLDH is not involved in the reduction of methylene-THF.

The glycine cleavage system is considered to be a loose complex composed of four proteins, which can catalyze the deamination, decarboxylation, and dehydrogenation of glycine to produce NH_3_, CO_2_, and NADH, while the methylene group combines with the one-carbon carrier THF to generate methylene-THF. Since DLDH is part of the glycine cleavage system as the L-protein (GsvL) (28), and *C. ljungdahlii* contains a sole DLDH coding gene (*lpd*A) in its genome, we predict that DLDH takes part in the reaction of the glycine cleavage system, which is also associated with the metabolism of methylene-THF. The MTHFR of *A. woodii* is not related to DLDH, although the genes which encode it are adjacent to an *lpd*A gene in the genome, as in *C. ljungdahlii*. A gene downstream of *lpd*A encodes an H-protein (GsvH), which also belongs to the glycine cleavage system, in both *C. ljungdahlii* (the *lpd*A and *gsv*H genes were separated by *acs/codh*) and *A. woodii*. The gene arrangement of *lpd*A and *met*FV may have evolved because MTHFR and the glycine cleavage system have a common substrate, methylene-THF, rather than because they catalyze a coupling reaction to reduce methylene-THF.

### Reduced Fd, Fld, and Trx could serve as electron donors for the reduction of methylene-THF in *C. ljungdahlii*.

**FIG 3:**
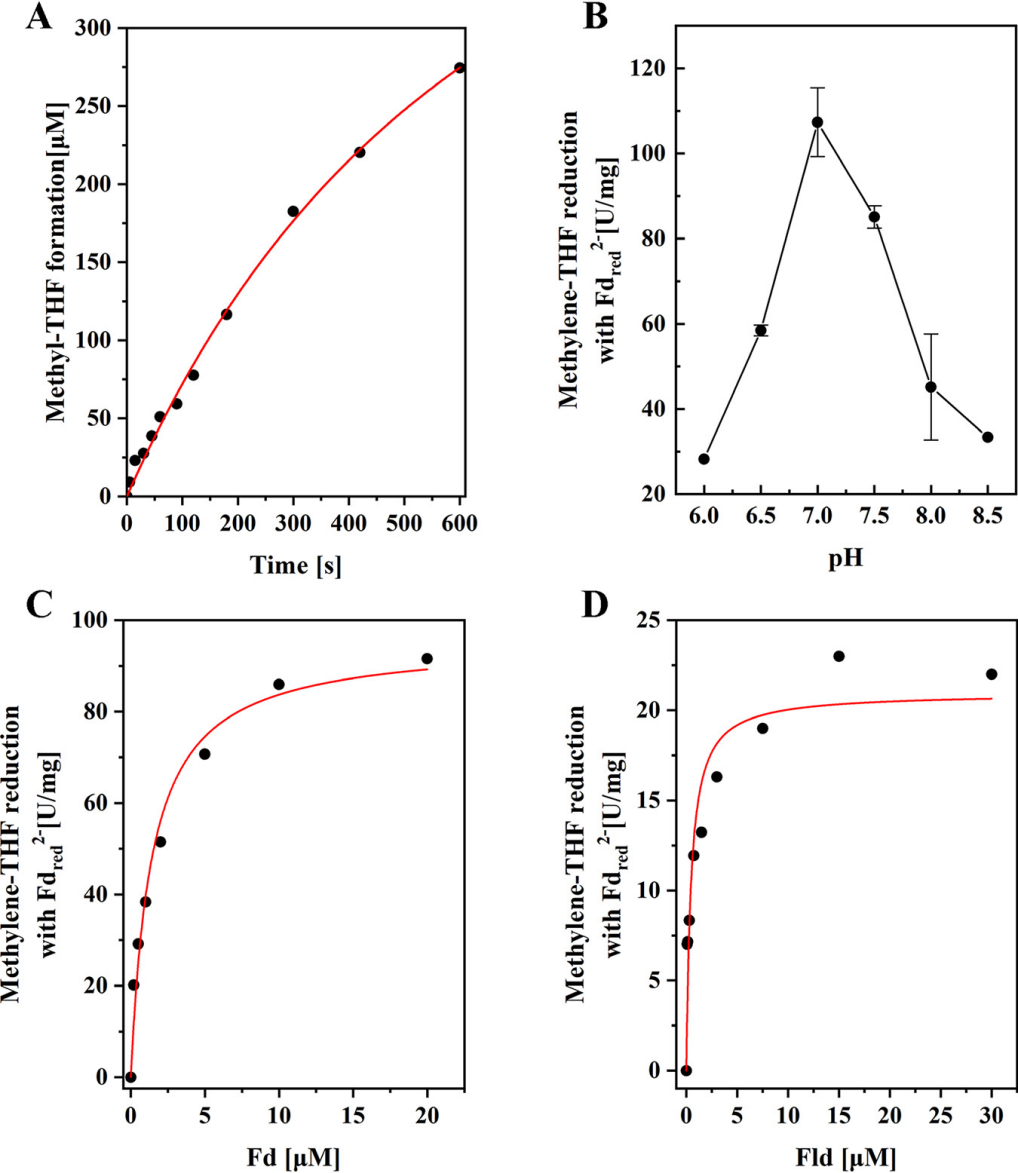
Kinetic properties of the purified MTHFR from *C. ljungdahlii*. The reaction mixture contained 50 mM Tris-HCl with 2 mM DTT and 5 μM FMN (pH 7.4), 0.5 mM methylene-THF, 10 μg CpI, and 50 μM Fd if not specified. The reaction was started by adding 0.62 μg MTHFR. The specific activity was calculated by monitoring the formation of methyl-THF using HPLC. (A) The kinetic curve of the methyl-THF formation with Fd_red_^2−^ as electron donor by MTHFR of *C. ljungdahlii*. (B) Effects of different pH on the reduction of methylene-THF with Fd_red_^2−^ by MTHFR. (C) Dependence of the reduction of methylene-THF with Fd_red_^2−^ on the Fd concentration. (D) Dependence of the reduction of methylene-THF with reduced Fld on the Fld concentration.

The reduced Fd, Fld, and Trx (Fd_red_^2−^, Fld_red_^2−^, and Trx_red_^2−^) from *C. ljungdahlii* were tested as electron donors, to measure the activity of the purified wild-type MTHFR. In the assays, the recombinant Fd and Fld were reduced by hydrogenase from C. pasteurianum (CpI) in a 100% H_2_ atmosphere, and the Trx was reduced with NADPH by TrxR. The reactions were monitored by regular sampling and detection of the formation of the product methyl-THF using HPLC, with which the substrate, methylene-THF, and the product, methyl-THF, could be determined (Fig. S4). It was found that Fd_red_^2−^, Fld_red_^2−^, and Trx_red_^2−^ could reduce methylene-THF catalyzed by MTHFR from *C. ljungdahlii.* Of these, MTHFR had the highest enzyme activity (91.2 U/mg) using Fd_red_^2−^ as an electron donor (Fig. 3A), much higher than that using Fld_red_^2−^ (22.1 U/mg) or Trx_red_^2−^ (7.4 U/mg). However, methyl-THF could not be detected when CpI or Fd was omitted. The optimal pH for the wild-type MTHFR activity with Fd_red_^2−^ was determined to be around 7.0, and the activity decreased to 30% at pH 6.0 or 8.5 (Fig. 3B). The measured *K_m_* values for Fd and Fld were very low (around 1.46 and 0.73 μM, respectively) (Fig. 3C and D). Compared with recent report on the Fd_red_^2−^-dependent activity (34.7 U/mg) and apparent *K_m_* value for Fd (9 μM) of the enzyme from Volker Müller’s group (31), the study presented a higher specific activity and the lower apparent *K_m_* value. This may be due to the use of Fd from *C. ljungdahlii* and Fd_red_^2−^ regeneration system in this study, which may be superior to the measurement of oxidation of Na_2_S_2_O_4_-reduced Fd used in the publication. These data indicate that Fd_red_^2−^ was the optimal electron donor for the MTHFR of *C. ljungdahlii*. Fld_red_^2−^ and Trx_red_^2−^ may also be used as electron donors under certain physiological conditions, such as iron-deficient environments.

## DISCUSSION

### Reduced Fd is probably the physiological electron donor for MTHFR in *C. ljungdahlii*.

MTHFR and the reactions it catalyzes in *C. ljungdahlii* have been poorly understood but are vital for understanding the maintenance of the redox balance and the energy metabolism of the WLP. The enzyme has been suggested to be an NADH-dependent or electron-bifurcating NADH- and Fd-dependent multimeric complex (16, 17, 19, 27, 42). However, the MTHFR purified from *C. ljungdahlii* was found in this study to be composed of only MetFV and could not catalyze the proposed reactions. This interpretation is supported by the fact that there is no gene in the genome of *C. ljungdahlii* that encodes an RnfC2- or HdrA-like subunit to form a complex with MetFV, as is the case in the NADH-dependent MTHFR complex from *A. woodii* and *M. thermoacetica*. We tested the ability of the NADH-dependent DLDH to act as an electron transport subunit and found the result to be negative, although its encoding gene *lpdA* is adjacent to *metFV* in the WLP gene cluster. This is in agreement with the fact that DLDH was separated through Q-Sepharose during the purification of MTHFR (Fig. 1A and Fig. S2D). We suggest that the DLDH functions as the L-protein of the glycine cleavage system, which is also associated with the metabolism of methylene-THF. It has also been proposed that EtfAB might couple with MTHFR to mediate an electron-bifurcating reaction (14, 16), a hypothesis which is based on the observation that EtfAB can form complexes with some dehydrogenases and reductases and catalyze electron-bifurcating reactions (43–46). However, although the genome of *C. ljungdahlii* carries five sets of *etfAB* genes, each set has its own neighboring dehydrogenase gene: one for butyryl-CoA dehydrogenase, one for the FixABCX complex, and others for lactate dehydrogenases. This organization suggests that there is small possibility of interaction of MetFV with a free EtfAB in *C. ljungdahlii*.

It has also been proposed that the physiological electron donor of MTHFR from *C. ljungdahlii* is a reducing equivalent equal to NADH, such as Fld or Trx (15). To investigate this hypothesis, we heterologously expressed the Fld and Trx reduction system of *C. ljungdahlii* and examined its activity. MTHFR could catalyze the reduction of methylene-THF with Fld_red_^2−^ with a specific activity of only a quarter of that of Fd_red_^2−^. Fld has been considered to be a substitute for Fd under certain extreme growth conditions, such as iron deficiency (40, 47). Although Fld has a higher redox potential (E_0_′ of −430, −60 mV) than Fd, the direct reduction of Fld by NAD(P)H has not been reported, suggesting that using Fld_red_^2−^ (reduced by H_2_ with a hydrogenase) as the electron donor of MTHFR cannot conserve extra energy compared with using Fd_red_^2−^. Trx_red_^2−^ could also serve as an electron donor for the reduction of methylene-THF, but its specific activity was much lower than that with Fd_red_^2−^. Considering that the transcription level of Fd gene is much higher than that of Fld and Trx in *C. ljungdahlii* (27, 48), Fd can be reduced in various reactions catalyzed by the enzymes such as CO dehydrogenase (grown on CO), HytA-E (grown on H_2_), and pyruvate:Fd oxidoreductases (grown on fructose), and the enzyme presented a high specific activity with Fd_red_^2−^ as electron donor and low apparent *K_m_* value for Fd, we therefore suggest that Fd_red_^2−^ might be the physiological electron donor for MTHFR in *C. ljungdahlii*, and Fld_red_^2−^ may be used as the electron donor under the iron-deficient conditions.

Although the redox potential for the Fd from *C. ljungdahlii* has not been determined, the redox potential of the clostridial type Fd is usually close to −400 mV under standard conditions and can probably reach −500 mV under physiological conditions (19). These levels are much lower than that of NADH (E_0_′ = −320 mV). Therefore, compared with NADH, the reduction of methylene-THF (E_0_′ = −200 mV) with Fd_red_^2−^ as an electron donor causes severe energy dissipation, which brings new challenges to the interpretation of the energy metabolism of *C. ljungdahlii*.

### MTHFRs are divided into five groups in bacteria.

**FIG 4:**
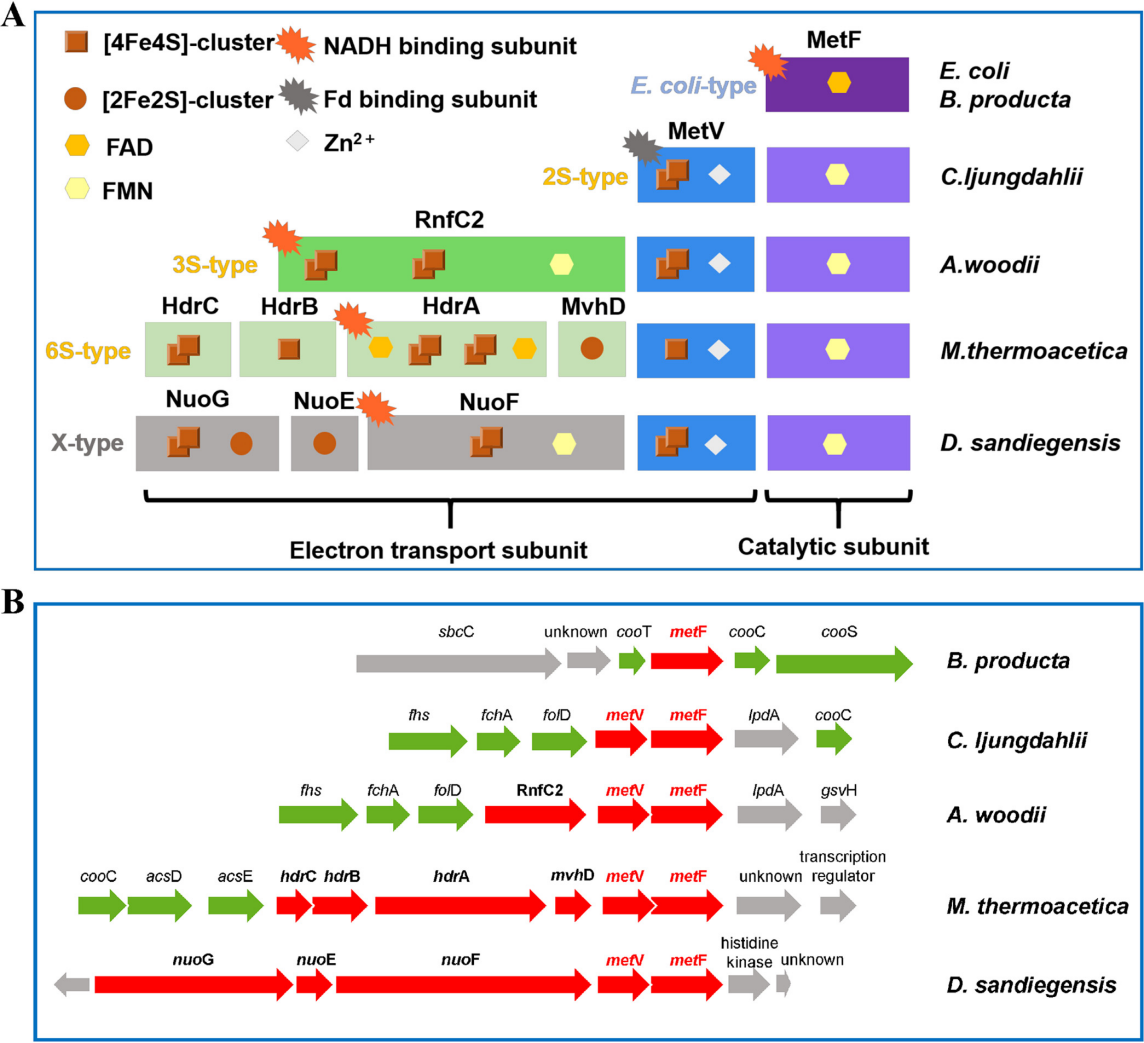
Schematic diagrams of typical subunit composition (A) and gene arrangement (B) of MTHFRs from different bacteria. (A) Based on the subunit composition, MTHFRs can be divided into four types: an E. coli type from aerobes and many anaerobic acetogens and three found only in strict anaerobes. (B) Typical gene arrangement of MTHFR in the WLP gene cluster of different acetogens. Red arrows, encoding genes of MTHFR from different acetogens; green arrows, WLP-related encoding genes; gray arrows, genes of WLP-unrelated or unknown function. *C. autoethanogenum*, *C. formicoaceticum*, C. carboxidivorans, and C. difficile have the same gene arrangement as *C. ljungdahlii*. *lpd*A, dihydrolipoamide dehydrogenase (DLDH); *gsv*H, glycine cleavage system H-protein; *fch*A, formyltetrahydrofolate cyclohydrolase; *fol*D, methylenetetrahydrofolate dehydrogenase; *fhs*, formate-THF ligase; *sbcC*, DNA repair exonuclease ATPase C subunit; *coo*T, *coo*S, *coo*C, *acs*D, and *acs*E, part of the CODH/ACS complex.

According to previous research (22, 31), we analyzed and compared the protein sequences and gene arrangements of the MTHFRs from different bacteria. Based on the subunit composition, MTHFRs can be classified into five groups (Fig. 4A). The simplest MTHFR, composed of only a single subunit of MetF, is widely present in aerobes. The structures of the type of MTHFR from E. coli and Thermus thermophilus have been resolved (35, 49). The type of MTHFR contains a binding site for FAD and catalyzes the reduction of methylene-THF with NADH through the ping-pong reaction mechanism (50). A flavin-lacking MTHFR was recently found in Mycobacterium smegmatis (32). Many anaerobic acetogens, including B. producta, Clostridium luticellarii, and Ruminococcus gauvreauii, also have E. coli-type MTHFRs. Of these, *B. producta* MTHFR has been shown to be O_2_ insensitive and has properties similar to those of E. coli MTHFR (20).

In anaerobic acetogens, the composition of most MTHFRs differs from that of the E. coli type. As well as MetF, they contain another electron transfer module, MetV, which is annotated as an iron-sulfur-zinc-containing protein. The MetFV-type MTHFRs can be divided into three subtypes: 2S (two subunits) type, 3S (three subunits) type, and 6S (six subunits) type. The MTHFR of *C. ljungdahlii* is a typical 2S type that is Fd_red_^2−^-dependent heterodimeric and binds an FMN to the MetF subunit. Another 2S type example is the *C. formicoaceticum* MTHFR, which has high protein sequence identities (74% for MetF and 68% for MetV) to the enzyme of *C. ljungdahlii*. However, it is a heterooctamer, binding an FAD to the MetF subunit (24). Recently, MetFV from *C. aceticum* and E. callanderi KIST612 has also been studied and has properties similar to those of MTHFR from *C. ljungdahlii*. Some acetogens, such as Clostridium drakei, Clostridium carboxidivorans, and *C. aceticum*, which have the same arrangements of the genes of the WLP as *C. ljungdahlii* (Fig. 4B), contain both E. coli-type and 2S-type MTHFR.

The representative 3S-type MTHFR is the enzyme from *A. woodii*, which contains an RnfC2 subunit as well as MetFV. The *A. woodii* MTHFR is NADH dependent, and its putative binding site for NADH is on the RnfC2 subunit (22). The RnfC2 subunit is composed of N-terminal MetV and C-terminal RnfC domains, which have 41% identity to the MetV subunit and the RnfC subunit, suggesting that *A. woodii* MTHFR might interact with the membrane-bound Rnf complex. *A. woodii* MTHFR showed a very low Fd_red_^2−^-dependent methylene-THF reduction activity. Its MetFV subunits show high sequence identities of 50% (MetF) and 37% (MetV) to the corresponding subunits from *C. ljungdahlii*.

The MTHFR from *M. thermoacetica* forms a complex of MetFV and heterodisulfide reductase modules of HdrABC-MvhD. This 6S-type MTHFR is an NADH-dependent heterohexamer, and its binding site for NADH is on HdrA (23). It has been proposed that the NADH-dependent methylene-THF reduction reaction is involved in flavin-based electron bifurcation, and the second electron acceptor is predicted to be different from Fd, which is functional for other electron-bifurcating enzymes (36, 43, 45, 51, 52). MetFV from *M. thermoacetica* also has high sequence identities (around 41% for both subunits) to those of *C. ljungdahlii*.

It is worth mentioning that we found another gene arrangement of MTHFR in Dethiosulfatarculus sandiegensis. The upstream of *metFV* contains the encoding genes of NuoEFG homolog, which is a common electron transfer module similar to HdrABC and EtfAB. This type of MTHFR exists widely in *Desulfobacterales* and *Chloroflexi*. Although it has not been studied, we speculate that it may be a new type of MTHFR (X type) with electron bifurcation function based on the knowledge of NuoEFG homolog as a module of the electron-bifurcating hydrogenase Hyt (51).

Genome analyses showed that MetFV-type MTHFR exists mainly in anaerobic bacteria. The *metF* and *metV* genes are conserved in MetFV-type homologs, and *metV* is located upstream of *metF* (Fig. 4B). In some species, like *M. thermoacetica*, the two genes overlap, suggesting that the two subunits may have coevolved. In acetogens, most of the adjacent genes of MTHFR are for the key enzymes in the WLP. Protein sequence alignment results show that the MetF subunit of *C. ljungdahlii* contains a conserved flavin binding site (Fig. S5A). The MetV subunit contains a conserved characteristic sequence, NGPCGG, called the MTHFR-C-terminal domain, and two [4Fe4S]-cluster binding sites (Fig. S5B). These features of MetFV are also conserved in other acetogenic bacteria. The conserved gene arrangement and high protein sequence identity suggest that MetFV-type MTHFRs are closely related evolutionarily. A phylogenetic analysis of MetFV-type MTHFRs from different bacteria was performed, with MetFV subunits as the targets. The result showed that MetFV-type MTHFRs were divided into four branches (Fig. S6), named groups I, II, III, and IV. The MTHFRs in group I, represented by *C. ljungdahlii*, are all 2S type, those in group II, represented by *A. woodii*, are all 3S-type, those in group III, represented by *D. sandiegensis*, are all X type, and those in group IV, represented by *M. thermoacetica*, are all composed of 6S-type MTHFRs. This further supports the classification of MTHFRs by subunit composition. From this point of view, the MTHFRs from *C. ljungdahlii* belong to 2S-type MTHFRs.

The various MetFV-type MTHFRs with different subunit compositions might have evolved in response to differences in energy metabolism of these anaerobic bacteria, especially in the three model acetogens *M. thermoacetica*, *A. woodii*, and *C. ljungdahlii*. Acetogens have been thought to be important players in the origin of life on Earth (53). They grow autotrophically on H_2_, CO_2_, or CO gas and conserve energy via an ancient mechanism of Fd-dependent electron bifurcation and anaerobic respiration with protons (Ech) or NAD^+^ (Rnf) as the electron acceptors at the thermodynamic limit of life (15, 19, 54). However, only the reaction catalyzed by 2S-type MTHFR represented by *C. ljungdahlii*, which reduces methylene-THF with Fd_red_^2−^ as the electron donor but not NADH like 3S- and 6S-type MTHFRs, is not economic in energy conservation, as mentioned above.

### Energy metabolism when *C. ljungdahlii* grows on CO or H_2_ plus CO_2_.

Previous discussions about the energy metabolism of *C. ljungdahlii* in publications have been based on the assumption that MTHFR is an NADH- and Fd-dependent electron-bifurcating enzyme or an NADH-dependent non-electron-bifurcating enzyme (16, 17, 19). In this work, we update this view, according to our new findings. The electron donors and acceptors of the key oxidoreductases of the WLP in *C. ljungdahlii* are predicted based on measurements in cell extracts of *C. autoethanogenum* (16, 51). The known mechanism for ATP synthesis in *C. ljungdahlii* depends mainly upon the membrane-associated Rnf-F_1_F_0_ATPase system. Since the C ring of Clostridium paradoxum was determined to contain 11 subunits (55, 56), a stoichiometry of 3.66 protons per ATP is generally used to calculate the yield of ATP in *C. ljungdahlii* (16, 27). The exact stoichiometry for ATP gain in *C. ljungdahlii* may be different, but it should not have a significant impact on the analysis of energy metabolism. In addition, the aldehyde:ferredoxin oxidoreductase (AOR) pathway is considered to be the main pathway for ethanol production (16, 17, 57), and the results of gene knockout experiments have also supported this interpretation (58). The following discussion focuses only on the AOR pathway.

**FIG 5:**
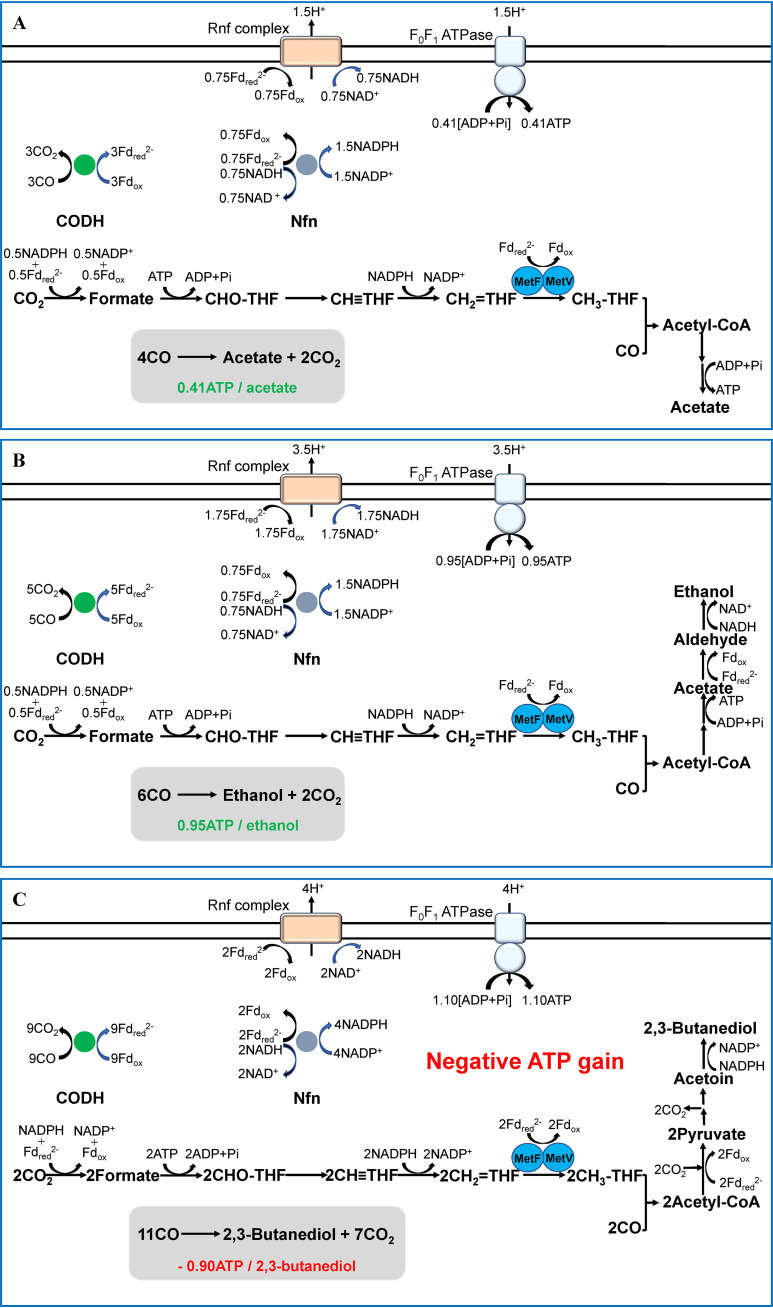
Schemes of the energy metabolism of *C. ljungdahlii* grown on CO, assuming that only acetate (A), ethanol (B), or 2,3-butanediol (C) is formed and that ethanol is produced via the reduction of acetic acid to acetaldehyde. The stoichiometry of 3.66 protons per mole ATP was used to calculate the yield of ATP, the value determined in *C. paradoxum*. The exact stoichiometry in *C. ljungdahlii*, which can be as low as 2.666 (mitochondria) and as high as 5 (cyanobacteria) based on the subunit number in C ring of F_0_F_1_ATPase, is not known, and independent of the exact stoichiometry, negative ATP gains are always obtained if not assuming ATP synthesis coupled to acetate reduction.

*C. ljungdahlii* ferments CO mainly to produce acetate, ethanol, and 2,3-butanediol. When only one product is assumed to be formed, the ATP yield per mol of product is 0.41, 0.95, or −0.9 mol for acetate, ethanol, or 2,3-butanediol, respectively (Fig. 5). The most abundant product in the fed-batch fermentation of CO is ethanol, followed by acetate and 2,3-butanediol, as demonstrated in a recent study (59). A high enough CO supply causes the cells to produce sufficient Fd_red_^2−^, which is helpful for the reduction of acetate by AOR and for the reduction of NAD^+^ by Rnf. These reactions can promote the AOR pathway and increase the generation of ethanol. Why does *C. ljungdahlii* form 2,3-butanediol with a negative ATP gain? The production of 2,3-butanediol occurs mainly in the stationary phase of cell growth, when the demand for ATP in cells decreases, and the release of excess reducing power in cells is more effective through the production of 2,3-butanediol.

When *C. ljungdahlii* grows on H_2_ plus CO_2_, the most abundantly produced metabolite is acetate, followed by ethanol (27, 58–60). When only ethanol is assumed to be formed, it yields 0.14 mol ATP per mol of ethanol produced (Fig. S7). However, when only acetate is assumed to be produced, 0.14 mol ATP is consumed per mol acetate formation. The establishment of this scheme is based on the following assumption: the Rnf-F_1_F_0_ATPase system can drive the reduction of Fd with NADH by hydrolyzing ATP (61), a reaction which has been confirmed in *A. woodii* (62). Since MTHFR uses Fd_red_^2−^ as the electron donor, it wastes more energy than previously hypothesized (15–17), leading to a negative ATP gain when *C. ljungdahlii* ferments H_2_ and CO_2_ to acetic acid. This finding is inconsistent with the fact that *C. ljungdahlii* grows well on H_2_ plus CO_2_. Öppinger et al. (31) put forward a hypothesis in a recent study to explain this confusion; that is, the Fd_red_^2−^-dependent methylene-THF reduction reaction may be coupled to other reactions, such as the one catalyzed by Rnf complex. This means that Fd_red_^2−^ acts as a driving force for proton pumping out of the membrane by the Rnf complex when reducing methylene-THF and further generates ATP through ATPase to conserve energy. Therefore, there could be a positive ATP gain when *C. ljungdahlii* grows on H_2_ and CO_2_. The hypothesis also applies to acetogenesis of other forms, such as the metabolism of Thermoanaerobacter kivui containing Ech and methanol metabolism of Eubacterium callanderi (31). However, this requires further research.

In addition, Song et al. (63) reported that the WLP can functionally cooperate with the glycine synthase-reductase pathway (GSRP) in CO_2_ fixation in a recent finding. This can happen in *C. ljungdahlii*, since it has complete GSRP-encoding genes. Using this mechanism, only one NADH and one NADPH are consumed from methylene-THF to acetyl phosphate through GSRP, while two Fd_red_^2−^ are required for the formation of acetyl phosphate from methylene-THF through WLP. Thus, more energy can be conserved through GSRP. However, since some acetogens, such as *C. autoethanogenum*, do not have GSRP-encoding genes, it is also possible that the 2S-type-MTHFR-containing acetogens represented by *C. ljungdahlii* have additional, currently unknown energy conservation mechanisms. Although it is not excluded that MTHFR from *C. ljungdahlii* has an unknown physiological electron donor, based on the high specific activity and low apparent *K_m_* value measured *in vitro* with Fd_red_^2−^ as an electron donor, we are more inclined to the idea that the Fd_red_^2−^-dependent reaction couples to other mechanisms such as Rnf-F_1_F_0_ATPase system to conserve energy. Therefore, further investigation is needed to establish the details of the mechanism of energy metabolism in this type of acetogen.

## MATERIALS AND METHODS

### Bacterial strains, culture conditions, and biochemicals.

*C. ljungdahlii* DSM 13528 and *C. pasteurianum* DSM 525 were obtained from the Deutsche Sammlung von Mikroorganismen und Zellkulturen GmbH, Braunschweig, Germany. E. coli C41(DE3) harboring the plasmids pCodonPlus and pRKISC was used for enhancing the expression of proteins, taking into account their codon usage and iron-sulfur cluster assembly (64).

*C. ljungdahlii* was cultivated anaerobically at 37°C with 2-liter glass bottles containing 1 liter of modified DSM medium 897 with a gas mixture (CO:CO_2_, 80:20) as the gas phase at 0.1 MPa. Fed-batch fermentation with pH control was carried out in a 5-liter bioreactor containing 2.5 liters of modified DSM medium 897. The supplied gas pressure with a headspace of a gas mixture (CO:CO_2_, 80:20) as the carbon source was controlled at 0.1 MPa, and the gas flow rate was 30 ml/min during the whole fermentation process (27, 59). The pH value was controlled at 6 automatically by adding 4 M KOH. *C. pasteurianum* was grown anaerobically at 37°C in a glucose-ammonium medium (38). The cells were harvested in the late-exponential phase. The culture was centrifuged at 6,000 × *g* for 10 min at 4°C. The pelleted cells were resuspended and washed in 30 ml of 50 mM Tris-HCl (pH 7.4) containing 2 mM dithiothreitol (DTT) and 10% glycerin (buffer A) and stored at −80°C under an atmosphere of 95% N_2_–5% H_2_.

NAD(P)^+^, NAD(P)H, FAD, FMN, methyl-THF, lysozyme, and BV were from Sigma-Aldrich (Shanghai, China). Methylene-THF was from Schircks Laboratories (Jona, Switzerland) or chemically produced from THF and formaldehyde.

Ferredoxin-dependent monomeric [FeFe]-hydrogenase from *C. pasteurianum* (CpI) was prepared as described previously (43, 65).

### Purification of MTHFR from *C. ljungdahlii*.

All steps were performed under strictly anoxic conditions at room temperature in an anaerobic chamber (Coy Laboratory, USA), which was filled with 95% N_2_–5% H_2_ and contained a palladium catalyst for O_2_ reduction with H_2_. About 6 g of frozen cells of *C. ljungdahlii* were suspended in 30 ml of buffer A containing 30 mg lysozyme and incubated at 37°C for 30 min. Then, the cell suspension was diluted to 50 ml and disrupted ultrasonically for 40 min in an ice-water bath. The ultrasonic breaker (Xinzhi, Ningbo, China) was set to work for 5 s and rest for 6 s for each cycle, and the power was set to 40%. Unbroken cells and cell debris were removed by centrifugation at 35,328 × *g* at 4°C for 40 min. The supernatant was used for enzyme purification. The activity of the reduction of BV with methyl-THF was measured.

The supernatant was first fractionated with gradient ammonium sulfate precipitation (25, 40, and 55% ammonium sulfate saturation). The MTHFR activity was found at the highest level in the supernatant with 55% saturation. After the removal of undissolved proteins by filtration, the fraction was loaded onto a high-performance (HP) phenyl-Sepharose column (2.6 by 10 cm) equilibrated with buffer A, containing 2.0 M ammonium sulfate. The protein was eluted in a gradient by gradually reducing the concentration of ammonium sulfate (2, 1.8, 1.6, 1.3, 1, 0.6, 0.3, and 0 M; each gradient elution was about 60 ml) with a flow rate of 3 ml/min. The MTHFR was eluted at a concentration of 1.3 M ammonium sulfate. The pooled fractions were concentrated and desalted using a 30 kDa-cutoff centrifugal filter. The concentrated sample was diluted about 30 times with buffer A and then applied to a Q-Sepharose column (HP, 1.6 by 2.5 cm, equilibrated with buffer A). The protein was eluted by gradually increasing the concentration of NaCl using buffer A containing 1 M NaCl (0, 0.1, 0.18, 0.25, 0.35, 0.50, 0.70, 0.85, and 1 M) at a flow rate of 1.5 ml/min. The MTHFR was eluted at a 0.18 M NaCl gradient. After being concentrated and desalted using a 30 kDa-cutoff centrifugal filter, the fraction was further loaded onto a DEAE-Sepharose column (Fast Flow, 1.6 by 2.5 cm). The elution method was similar to that of Q-Sepharose column with a gradient of NaCl concentration (0, 0.08, 0.16, 0.24, 0.35, 0.70, and 1 M), where the elution buffer was adjusted to pH 8. The MTHFR was recovered in a peak eluted around 0.16 M NaCl. The fraction was concentrated and desalted with a centrifugal filter and then stored at −20°C in buffer A under an atmosphere of 95% N_2_–5% H_2_ until used.

### Heterologous expression and purification of MetFV, DLDH, Fd, Fld, Trx, and TrxR.

The genes were amplified using PCR, with the genomic DNA of *C. ljungdahlii* as a template. The primers used are listed in Table 2. The His tags were added to both N and C termini of MetFV and Fld and the N terminus of DLDH, Fd, Trx, and TrxR. All genes were cloned into pET-28b plasmids at the restriction sites shown in the primers listed in Table 2, and the recombinant constructs were transformed into E. coli C41 (DE3), except pET-28b*-fld*, which was transformed into E. coli BL21(DE3)-groELS. This host can overexpress the molecular chaperone GroELS to promote protein folding. Before inoculation, the medium was supplemented with kanamycin (25 mg liter^−1^), chloramphenicol (12.5 mg liter^−1^), and tetracycline (5 mg liter^−1^) for E. coli C41(DE3) and only kanamycin (50 mg liter^−1^) for E. coli BL21(DE3)-groELS, to maintain the plasmids. Fe and S sources (0.12 g cysteine, 0.1 g FeSO_4_, and 0.2 g ferric ammonium citrate per liter culture) were added to enhance iron-sulfur cluster synthesis for MetFV and Fd expression. The recombinant cells were grown aerobically in 1 liter of Terrific broth at 37°C. When an optical density at 600 nm (OD_600_) of 0.8 was reached, 0.5 mM isopropyl-β-d-thiogalactoside was added to the culture, and the cells were grown at 25°C for 14 h. The cells were harvested by centrifugation at 6,000 × *g* at 4°C and washed with 50 mM sodium phosphate (pH 7.0).

**TABLE 2:** Primers used in this study

Primer	Primer sequence (5′→3′)^*a*^
*metFV-*F	CTAGCTAGCATGATTATTTCAGAAAAAAAATCT (NheI)
*metFV-*R	CCGCTCGAGTAATCCTGCTTCATCCAAT (XhoI)
*fd*-F	CATGCCATGG CATATAAAATTACAGAGGAT (NcoI)
*fd*-R	CCGCTCGAGGCTTTCTTCAACTGGTGCT (XhoI)
*trx*-F	CATGCCATGGTAGAGGAAATAAAAGATCAAG (NcoI)
*trx*-R	CCGCTCGAGTATATGTTTTTTCAAAGCATCTTTT (XhoI)
*trxr*-F	CATGCCATGGACACTAGATATGATATTGCTATA (NcoI)
*trxr*-R	CCGCTCGAGATTTTTATCAATGTAAAAGACAGCA (XhoI)
*dldh*-F	CTAGCTAGCATGAAATTAGTTGTAATTGGTGG (NheI)
*dldh*-R	CCGCTCGAGTTAATCAACAGAATGAACACATT (XhoI)
*fld*-F	CTAGCTAGCATGAAAAAAATATCAATTATTTATTGGAGTGGT (NheI)
*fld*-R	CCGCTCGAG TTTTTCTGCTAATGCCTCGC (XhoI)

a The recognition site is underlined.

The cell extracts were prepared using the same method as that used for *C. ljungdahlii*. The supernatant was loaded onto a 5 ml HisTrap column (GE Healthcare, Buckinghamshire, UK) that had previously been equilibrated with binding buffer (20 mM sodium phosphate containing 0.5 M NaCl and 2 mM DTT [pH 7.4], 5 μM FMN was added for MetFV and 5 μM FAD was added for DLDH). The target proteins were eluted at different concentrations of imidazole (100 to 200 mM for MTHFR and TrxR, 75 mM for Fd and Fld, and 200 mM for Trx) using a stepwise elution gradient. The proteins were washed with 50 mM Tris-HCl (pH 7.4) using centrifugal filters and were stored at −20°C until used.

### Enzyme assays.

Enzyme activities were measured at 37°C in 1.5 ml anoxic cuvettes closed with a rubber stopper under an H_2_ or N_2_ atmosphere, as indicated. The buffer containing 50 mM Tris-HCl (pH 7.4), 2 mM DTT, and 10 μM FMN was used as the basic assay mixture. The addition of FMN in the assay is to supplement the FMN that may fall off during the purification process. The reactions were monitored spectrophotometrically or by determining product formation using HPLC. When detected photometrically, BV reduction was monitored at 555 nm (ε = 12.1 mM^−1 ^cm^−1^), the reduction of NAD(P)^+^ or the oxidation of NAD(P)H was monitored at 340 nm (ε = 6.2 mM^−1 ^cm^−1^), and Fd reduction was monitored at 430 nm (Δε_ox-red_ ≈ 13.1 mM^−1 ^cm^−1^) (23, 36). When Fd_red_^2−^, Fld_red_^2−^, or Trx_red_^2−^ was used to reduce methylene-THF, the reactions were monitored by determining the formation of methyl-THF using HPLC. One unit (U) equals the formation of 1 μmol of product or the consumption of 1 μmol of substrate per min. During the measurements, the cuvettes, vials, and rubber stoppers for BV-related assays were not used for other assays, to avoid interference caused by trace amounts of BV absorbed on these items.

When the oxidation of methyl-THF with BV was monitored, the basic assay mixture was supplemented with 20 mM BV and 1 mM methyl-THF under a N_2_ atmosphere. In the initial purification step, the determination of BV reduction is affected by hydrogenase activity in the samples, so it was necessary to start the reaction with methyl-THF.

When the reduction of methylene-THF with NAD(P)H was followed, the basic assay mixture was supplemented with 0.5 mM methylene-THF, 0.1 mM NADH or NADPH, 1 mM NAD^+^ or NADP^+^, or 50 μM Fd. The gas phase was 100% N_2_. When the coupling reaction of MTHFR and DLDH was measured, 1 mM lipoamide was added, and the reaction was monitored using HPLC to measure the formation of methyl-THF.

When the reduction of methylene-THF with Fd_red_^2−^ and Fld_red_^2−^ was measured, the basic assay mixture was supplemented with 0.5 mM methylene-THF and 50 μM Fd or Fld, and then 3 U of CpI hydrogenase from *C. pasteurianum* was added to reduce Fd and Fld, under a 100% H_2_ atmosphere. When Trx_red_^2−^ was used as the electron donor, 1 mM NADPH, 100 μM Trx, and 50 μg TrxR were added under a 100% N_2_ atmosphere. These reaction conditions were equivalent to regenerating systems of redox proteins. After incubation at 37°C for 5 min, an aliquot was taken using a gas-tight syringe as a control. For the assays, MTHFR was added to start the reaction, and aliquots were sampled at specified intervals. A double volume of water free ethanol was added immediately to the sample to inactivate the enzyme and stop the reaction. Then, the samples were centrifuged at 15,000 × *g* and 4°C for 10 min. The sample was then diluted 20 times and filtered for HPLC analysis. For the determination of the optimum pH, buffers with different pHs were used: 50 mM potassium phosphate for pH 6.0 to 8.0 and 50 mM Tris-HCl for pH 8.5.

### Analytical methods.

Protein concentration was determined using Bradford analysis with bovine serum albumin as a standard (66). The protein densitometry analysis was performed using ExPASy-Compute pI/*M*_w_ tool (https://web.expasy.org/compute_pi/). The proteins were separated by SDS-PAGE using 12.5% PAGE gel fast preparation kits (EpiZyme, Shanghai, China) and stained with Coomassie brilliant blue G250. Native PAGE and activity staining were performed in the anaerobic chamber mentioned above, which was filled with 95% N_2_–5% H_2_. In the gel preparation, sample treatment, and electrophoresis, SDS and the reducing agents were omitted. The activity staining solution contained 50 mM potassium phosphate buffer (pH 7.3), 10 mM BV, and 0.5 mM methyl-THF. The molecular mass of the MTHFR was determined by gel filtration on a GE Superdex G200 column (10 by 300 mm), calibrated with protein standards (Fig. S2D). The buffer was 50 mM Tris-HCl (pH 7.4) containing 150 mM NaCl and 2 mM DTT, and the flow rate was set at 0.5 ml min^−1^. The proteins were identified by peptide mass fingerprinting analysis (BGI, Beijing, China). The proteins were first reduced and alkylated by DTT and iodoacetamide and then were digested by trypsin. The peptides were analyzed using HPLC prominence nano 2D (Shimazhu, Japan) and C_18_ (5 μm, 150A, Eprogen) coupled to an electrospray ionization-quadrupole time of flight mass spectrometer MicrOTOF QII (Bruker Daltonics). Acquisition of mass spectra and fragmentation were based on the intensity and charge state of the precursor ions, and the mass lists were matched to a custom proteome database using the Mascot search engine (version 2.3.01; Matrix Sciences).

The reaction substrates and products were quantified using a Shimadzu HPLC system (67), which was equipped with a CBM system controller, SIL autosampler, LC-20AT pump, CTO column oven, and RF fluorescence detector. The reactants were separated with a ChromCore 120 C_18_ column (250 by 4.6 mm). The column temperature was maintained at 30°C. The excitation and emission wavelengths for the fluorescence detection were set at 290 and 356 nm, respectively. A mixture of 33 mM potassium phosphate buffer (pH 3.0) containing 7.0% acetonitrile was used as the mobile phase to separate the reactants, and the flow rate was set at 0.5 ml/min. A standard curve of methyl-THF and methylene-THF was prepared for quantification.

The flavin content of the enzymes was measured using HPLC, as described previously (68). The iron content was determined colorimetrically with ferene (69).

## References

[1] Müller V. 2003. Energy conservation in acetogenic bacteria. Appl Environ Microbiol 69:6345–6353. doi:10.1128/AEM.69.11.6345-6353.2003. 14602585 PMC262307

[2] Wilkins MR, Atiyeh HK. 2011. Microbial production of ethanol from carbon monoxide. Curr Opin Biotechnol 22:326–330. doi:10.1016/j.copbio.2011.03.005. 21470845

[3] Köpke M, Mihalcea C, Liew F, Tizard JH, Ali MS, Conolly JJ, Al-Sinawi B, Simpson SD. 2011. 2,3-Butanediol production by acetogenic bacteria, an alternative route to chemical synthesis, using industrial waste gas. Appl Environ Microbiol 77:5467–5475. doi:10.1128/AEM.00355-11. 21685168 PMC3147483

[4] Zhang L, Zhao R, Jia D, Jiang W, Gu Y. 2020. Engineering *Clostridium ljungdahlii* as the gas-fermenting cell factory for the production of biofuels and biochemicals. Curr Opin Chem Biol 59:54–61. doi:10.1016/j.cbpa.2020.04.010. 32480247

[5] Poehlein A, Schmidt S, Kaster AK, Goenrich M, Vollmers J, Thurmer A, Bertsch J, Schuchmann K, Voigt B, Hecker M, Daniel R, Thauer RK, Gottschalk G, Müller V. 2012. An ancient pathway combining carbon dioxide fixation with the generation and utilization of a sodium ion gradient for ATP synthesis. PLoS One 7:e33439. doi:10.1371/journal.pone.0033439. 22479398 PMC3315566

[6] Ragsdale SW, Pierce E. 2008. Acetogenesis and the Wood-Ljungdahl pathway of CO_2_ fixation. Biochim Biophys Acta 1784:1873–1898. doi:10.1016/j.bbapap.2008.08.012. 18801467 PMC2646786

[7] Ragsdale SW. 2008. Enzymology of the Wood-Ljungdahl pathway of acetogenesis. Ann N Y Acad Sci 1125:129–136. doi:10.1196/annals.1419.015. 18378591 PMC3040112

[8] Ljungdahl LG. 1986. The autotrophic pathway of acetate synthesis in acetogenic bacteria. Annu Rev Microbiol 40:415–450. doi:10.1146/annurev.mi.40.100186.002215. 3096193

[9] Biegel E, Müller V. 2010. Bacterial Na^+^-translocating ferredoxin:NAD^+^ oxidoreductase. Proc Natl Acad Sci USA 107:18138–18142. doi:10.1073/pnas.1010318107. 20921383 PMC2964206

[10] Schoelmerich MC, Müller V. 2019. Energy conservation by a hydrogenase-dependent chemiosmotic mechanism in an ancient metabolic pathway. Proc Natl Acad Sci USA 116:6329–6334. doi:10.1073/pnas.1818580116. 30850546 PMC6442639

[11] Buckel W, Thauer RK. 2018. Flavin-based electron bifurcation, a new mechanism of biological energy coupling. Chem Rev 118:3862–3886. doi:10.1021/acs.chemrev.7b00707. 29561602

[12] Peters JW, Beratan DN, Bothner B, Dyer RB, Harwood CS, Heiden ZM, Hille R, Jones AK, King PW, Lu Y, Lubner CE, Minteer SD, Mulder DW, Raugei S, Schut GJ, Seefeldt LC, Tokmina-Lukaszewska M, Zadvornyy OA, Zhang P, Adams MW. 2018. A new era for electron bifurcation. Curr Opin Chem Biol 47:32–38. doi:10.1016/j.cbpa.2018.07.026. 30077080 PMC9113080

[13] Müller V, Chowdhury NP, Basen M. 2018. Electron bifurcation: a long-hidden energy-coupling mechanism. Annu Rev Microbiol 72:331–353. doi:10.1146/annurev-micro-090816-093440. 29924687

[14] Köpke M, Held C, Hujer S, Liesegang H, Wiezer A, Wollherr A, Ehrenreich A, Liebl W, Gottschalk G, Dürre P. 2010. *Clostridium ljungdahlii* represents a microbial production platform based on syngas. Proc Natl Acad Sci USA 107:13087–13092. doi:10.1073/pnas.1004716107. 20616070 PMC2919952

[15] Schuchmann K, Müller V. 2014. Autotrophy at the thermodynamic limit of life: a model for energy conservation in acetogenic bacteria. Nat Rev Microbiol 12:809–821. doi:10.1038/nrmicro3365. 25383604

[16] Mock J, Zheng Y, Mueller AP, Ly S, Tran L, Segovia S, Nagaraju S, Köpke M, Dürre P, Thauer RK. 2015. Energy conservation associated with ethanol formation from H_2_ and CO_2_ in *Clostridium autoethanogenum* involving electron bifurcation. J Bacteriol 197:2965–2980. doi:10.1128/JB.00399-15. 26148714 PMC4542177

[17] Katsyv A, Müller V. 2020. Overcoming energetic barriers in acetogenic C1 conversion. Front Bioeng Biotechnol 8:621166. doi:10.3389/fbioe.2020.621166. 33425882 PMC7793690

[18] Thauer RK, Jungermann K, Decker K. 1977. Energy conservation in chemotrophic anaerobic bacteria. Bacteriol Rev 41:100–180. doi:10.1128/br.41.1.100-180.1977. 860983 PMC413997

[19] Buckel W, Thauer RK. 2013. Energy conservation via electron bifurcating ferredoxin reduction and proton/Na^+^ translocating ferredoxin oxidation. Biochim Biophys Acta 1827:94–113. doi:10.1016/j.bbabio.2012.07.002. 22800682

[20] Wohlfarth G, Geerligs G, Diekert G. 1990. Purification and properties of a NADH-dependent 5,10-methylenetetrahydrofolate reductase from *Peptostreptococcus productus*. Eur J Biochem 192:411–417. doi:10.1111/j.1432-1033.1990.tb19242.x. 2209595

[21] Sheppard CA, Trimmer EE, Matthews RG. 1999. Purification and properties of NADH-dependent 5,10-methylenetetrahydrofolate reductase (MetF) from *Escherichia coli*. J Bacteriol 181:718–725. doi:10.1128/JB.181.3.718-725.1999. 9922232 PMC93435

[22] Bertsch J, Oppinger C, Hess V, Langer JD, Müller V. 2015. Heterotrimeric NADH-oxidizing methylenetetrahydrofolate reductase from the acetogenic bacterium *Acetobacterium woodii*. J Bacteriol 197:1681–1689. doi:10.1128/JB.00048-15. 25733614 PMC4403655

[23] Mock J, Wang S, Huang H, Kahnt J, Thauer RK. 2014. Evidence for a hexaheteromeric methylenetetrahydrofolate reductase in *Moorella thermoacetica*. J Bacteriol 196:3303–3314. doi:10.1128/JB.01839-14. 25002540 PMC4135698

[24] Clark JE, Ljungdahl LG. 1984. Purification and properties of 5,10-methylenetetrahydrofolate reductase, an iron-sulfur flavoprotein from *Clostridium formicoaceticum*. J Biol Chem 259:10845–10849. doi:10.1016/S0021-9258(18)90590-9. 6381490

[25] Wiechmann A, Müller V. 2021. Energy conservation in the acetogenic bacterium *Clostridium aceticum*. Microorganisms 9. doi:10.3390/microorganisms9020258. PMC7911925 33513854

[26] Dietrich HM, Kremp F, Oppinger C, Ribaric L, Muller V. 2021. Biochemistry of methanol-dependent acetogenesis in *Eubacterium callanderi* KIST612. Environ Microbiol 23:4505–4517. doi:10.1111/1462-2920.15643. 34125457

[27] Liu Z-Y, Jia D-C, Zhang K-D, Zhu H-F, Zhang Q, Jiang W-H, Gu Y, Li F-L. 2020. Ethanol metabolism dynamics in *Clostridium ljungdahlii* grown on carbon monoxide. Appl Environ Microbiol 86:e00730-20. doi:10.1128/AEM.00730-20. 32414802 PMC7357473

[28] Kikuchi G, Motokawa Y, Yoshida T, Hiraga K. 2008. Glycine cleavage system: reaction mechanism, physiological significance, and hyperglycinemia. Proc Jpn Acad, Ser B 84:246–263. doi:10.2183/pjab.84.246. 18941301 PMC3666648

[29] de Kok A, Kornfeld S, Benziman M, Milner Y. 1980. Subunit composition and partial reaction of 2-oxoglutarate dehydrogenase complex of *Acetobacter xylinum*. Eur J Biochem 106:49–58. doi:10.1111/j.1432-1033.1980.tb05996.x. 6896181

[30] Arner ES, Holmgren A. 2000. Physiological functions of thioredoxin and thioredoxin reductase. Eur J Biochem 267:6102–6109. doi:10.1046/j.1432-1327.2000.01701.x. 11012661

[31] Öppinger C, Kremp F, Müller V. 2021. Is reduced ferredoxin the physiological electron donor for MetVF-type methylenetetrahydrofolate reductases in acetogenesis? A hypothesis. Int Microbiol doi:10.1007/s10123-021-00190-0. PMC8760232 34255221

[32] Sah S, Lahry K, Talwar C, Singh S, Varshney U. 2020. Monomeric NADH-oxidizing methylenetetrahydrofolate reductases from *Mycobacterium smegmatis* lack flavin coenzyme. J Bacteriol 202:e00709-19. doi:10.1128/JB.00709-19. 32253341 PMC7253612

[33] Daubner SC, Matthews RG. 1982. Purification and properties of methylenetetrahydrofolate reductase from pig liver. J Biol Chem 257:140–144. doi:10.1016/S0021-9258(19)68337-7. 6975779

[34] Zhou J, Kang SS, Wong PW, Fournier B, Rozen R. 1990. Purification and characterization of methylenetetrahydrofolate reductase from human cadaver liver. Biochem Med Metab Biol 43:234–242. doi:10.1016/0885-4505(90)90029-Z. 2383427

[35] Igari S, Ohtaki A, Yamanaka Y, Sato Y, Yohda M, Odaka M, Noguchi K, Yamada K. 2011. Properties and crystal structure of methylenetetrahydrofolate reductase from *Thermus thermophilus* HB8. PLoS One 6:e23716. doi:10.1371/journal.pone.0023716. 21858212 PMC3156243

[36] Wang S, Huang H, Moll J, Thauer RK. 2010. NADP^+^ reduction with reduced ferredoxin and NADP^+^ reduction with NADH are coupled via an electron-bifurcating enzyme complex in *Clostridium kluyveri*. J Bacteriol 192:5115–5123. doi:10.1128/JB.00612-10. 20675474 PMC2944534

[37] Yang SS, Ljungdahl LG, LeGall J. 1977. A four-iron, four-sulfide ferredoxin with high thermostability from *Clostridium thermoaceticum*. J Bacteriol 130:1084–1090. doi:10.1128/jb.130.3.1084-1090.1977. 863852 PMC235330

[38] Scherer PA, Thauer RK. 1978. Purification and properties of reduced ferredoxin: CO_2_ oxidoreductase from *Clostridium pasteurianum*, a molybdenum iron-sulfur-protein. Eur J Biochem 85:125–135. doi:10.1111/j.1432-1033.1978.tb12220.x. 639811

[39] Romero A, Caldeira J, Legall J, Moura I, Moura JJ, Romao M. 1996. Crystal structure of flavodoxin from *Desulfovibrio desulfuricans* ATCC 27774 in two oxidation states. Eur J Biochem 239:190–196. doi:10.1111/j.1432-1033.1996.0190u.x. 8706707

[40] Chowdhury NP, Klomann K, Seubert A, Buckel W. 2016. Reduction of flavodoxin by electron bifurcation and sodium ion-dependent reoxidation by NAD^+^ catalyzed by ferredoxin-NAD^+^ reductase (Rnf). J Biol Chem 291:11993–12002. doi:10.1074/jbc.M116.726299. 27048649 PMC4933252

[41] Harms C, Meyer MA, Andreesen J. 1998. Fast purification of thioredoxin reductases and of thioredoxins with an unusual redox-active centre from anaerobic, amino-acid-utilizing bacteria. Microbiology (Reading SGM) 144 (Pt 3):793–800. doi:10.1099/00221287-144-3-793. 9534247

[42] Valgepea K, de Souza Pinto Lemgruber R, Meaghan K, Palfreyman RW, Abdalla T, Heijstra BD, Behrendorff JB, Tappel R, Köpke M, Simpson SD, Nielsen LK, Marcellin E. 2017. Maintenance of ATP homeostasis triggers metabolic shifts in gas-fermenting acetogens. Cell Syst 4:505–515.e5. doi:10.1016/j.cels.2017.04.008. 28527885

[43] Li F, Hinderberger J, Seedorf H, Zhang J, Buckel W, Thauer RK. 2008. Coupled ferredoxin and crotonyl coenzyme A (CoA) reduction with NADH catalyzed by the butyryl-CoA dehydrogenase/Etf complex from *Clostridium kluyveri*. J Bacteriol 190:843–850. doi:10.1128/JB.01417-07. 17993531 PMC2223550

[44] Chowdhury NP, Mowafy AM, Demmer JK, Upadhyay V, Koelzer S, Jayamani E, Kahnt J, Hornung M, Demmer U, Ermler U, Buckel W. 2014. Studies on the mechanism of electron bifurcation catalyzed by electron transferring flavoprotein (Etf) and butyryl- CoA dehydrogenase(Bcd) of *Acidaminococcus fermentans*. J Biol Chem 289:5145–5157. doi:10.1074/jbc.M113.521013. 24379410 PMC3931072

[45] Bertsch J, Parthasarathy A, Buckel W, Müller V. 2013. An electron-bifurcating caffeyl-CoA reductase. J Biol Chem 288:11304–11311. doi:10.1074/jbc.M112.444919. 23479729 PMC3630892

[46] Weghoff MC, Bertsch J, Müller V. 2015. A novel mode of lactate metabolism in strictly anaerobic bacteria. Environ Microbiol 17:670–677. doi:10.1111/1462-2920.12493. 24762045

[47] Ragsdale SW, Ljungdahl LG. 1984. Characterization of ferredoxin, flavodoxin, and rubredoxin from *Clostridium formicoaceticum* grown in media with high and low iron contents. J Bacteriol 157:1–6. doi:10.1128/jb.157.1.1-6.1984. 6690418 PMC215120

[48] Aklujkar M, Leang C, Shrestha PM, Shrestha M, Lovley DR. 2017. Transcriptomic profiles of *Clostridium ljungdahlii* during lithotrophic growth with syngas or H_2_ and CO_2_ compared to organotrophic growth with fructose. Sci Rep 7:13135. doi:10.1038/s41598-017-12712-w. 29030620 PMC5640608

[49] Guenther BD, Sheppard CA, Tran P, Rozen R, Matthews RG, Ludwig ML. 1999. The structure and properties of methylenetetrahydrofolate reductase from *Escherichia coli* suggest how folate ameliorates human hyperhomocysteinemia. Nat Struct Biol 6:359–365. doi:10.1038/7594. 10201405

[50] Pejchal R, Sargeant R, Ludwig ML. 2005. Structures of NADH and CH_3_-H_4_folate complexes of *Escherichia coli* methylenetetrahydrofolate reductase reveal a spartan strategy for a ping-pong reaction. Biochemistry 44:11447–11457. doi:10.1021/bi050533q. 16114881

[51] Wang S, Huang H, Kahnt J, Mueller AP, Kopke M, Thauer RK. 2013. NADP-specific electron-bifurcating [FeFe]-hydrogenase in a functional complex with formate dehydrogenase in *Clostridium autoethanogenum* grown on CO. J Bacteriol 195:4373–4386. doi:10.1128/JB.00678-13. 23893107 PMC3807470

[52] Wang S, Huang H, Kahnt J, Thauer RK. 2013. A reversible electron-bifurcating ferredoxin- and NAD-dependent [FeFe]-hydrogenase (HydABC) in *Moorella thermoacetica*. J Bacteriol 195:1267–1275. doi:10.1128/JB.02158-12. 23316038 PMC3591994

[53] Martin WF, Sousa FL, Lane N. 2014. Evolution. Energy at life’s origin. Science 344:1092–1093. doi:10.1126/science.1251653. 24904143

[54] Buckel W, Thauer RK. 2018. Flavin-based electron bifurcation, ferredoxin, flavodoxin, and anaerobic respiration with protons (Ech) or NAD^+^ (Rnf) as electron acceptors: a historical review. Front Microbiol 9:401. doi:10.3389/fmicb.2018.00401. 29593673 PMC5861303

[55] Ferguson SA, Keis S, Cook GM. 2006. Biochemical and molecular characterization of a Na^+^-translocating F1Fo-ATPase from the thermoalkaliphilic bacterium *Clostridium paradoxum*. J Bacteriol 188:5045–5054. doi:10.1128/JB.00128-06. 16816177 PMC1539966

[56] Meier T, Ferguson SA, Cook GM, Dimroth P, Vonck J. 2006. Structural investigations of the membrane-embedded rotor ring of the F-ATPase from *Clostridium paradoxum*. J Bacteriol 188:7759–7764. doi:10.1128/JB.00934-06. 16980459 PMC1636304

[57] Nissen LS, Basen M. 2019. The emerging role of aldehyde:ferredoxin oxidoreductases in microbially-catalyzed alcohol production. J Biotechnol 306:105–117. doi:10.1016/j.jbiotec.2019.09.005. 31541665

[58] Liew F, Henstra AM, Kӧpke M, Winzer K, Simpson SD, Minton NP. 2017. Metabolic engineering of *Clostridium autoethanogenum* for selective alcohol production. Metab Eng 40:104–114. doi:10.1016/j.ymben.2017.01.007. 28111249 PMC5367853

[59] Zhu HF, Liu ZY, Zhou X, Yi JH, Lun ZM, Wang SN, Tang WZ, Li FL. 2020. Energy conservation and carbon flux distribution during fermentation of CO or H_2_/CO_2_ by *Clostridium ljungdahlii*. Front Microbiol 11:416. doi:10.3389/fmicb.2020.00416. 32256473 PMC7092622

[60] Emerson DF, Woolston BM, Liu N, Donnelly M, Currie DH, Stephanopoulos G. 2019. Enhancing hydrogen-dependent growth of and carbon dioxide fixation by *Clostridium ljungdahlii* through nitrate supplementation. Biotechnol Bioeng 116:294–306. doi:10.1002/bit.26847. 30267586

[61] Tremblay PL, Zhang T, Dar SA, Leang C, Lovley DR. 2012. The Rnf complex of *Clostridium ljungdahlii* is a proton-translocating ferredoxin:NAD^+^ oxidoreductase essential for autotrophic growth. mBio 4:e00406-12. doi:10.1128/mBio.00406-12. 23269825 PMC3531802

[62] Hess V, Schuchmann K, Müller V. 2013. The ferredoxin:NAD^+^ oxidoreductase (Rnf) from the acetogen *Acetobacterium woodii* requires Na^+^ and is reversibly coupled to the membrane potential. J Biol Chem 288:31496–31502. doi:10.1074/jbc.M113.510255. 24045950 PMC3814746

[63] Song Y, Lee JS, Shin J, Lee GM, Jin S, Kang S, Lee JK, Kim DR, Lee EY, Kim SC, Cho S, Kim D, Cho BK. 2020. Functional cooperation of the glycine synthase-reductase and Wood-Ljungdahl pathways for autotrophic growth of *Clostridium drakei*. Proc Natl Acad Sci USA 117:7516–7523. doi:10.1073/pnas.1912289117. 32170009 PMC7132306

[64] Nakamura M, Saeki K, Takahashi Y. 1999. Hyperproduction of recombinant ferredoxins in *Escherichia coli* by coexpression of the ORF1-ORF2-iscS-iscU-iscA-hscB-hs cA-fdx-ORF3 gene cluster. J Biochem 126:10–18. doi:10.1093/oxfordjournals.jbchem.a022409. 10393315

[65] Chen JS, Mortenson LE. 1974. Purification and properties of hydrogenase from *Clostridium pasteurianum* W5. Biochim Biophys Acta 371:283–298. doi:10.1016/0005-2795(74)90025-7. 4433567

[66] Bradford MM. 1976. A rapid and sensitive method for the quantitation of microgram quantities of protein utilizing the principle of protein-dye binding. Anal Biochem 72:248–254. doi:10.1006/abio.1976.9999. 942051

[67] Huang L, Zhang J, Hayakawa T, Tsuge H. 2001. Assays of methylenetetrahydrofolate reductase and methionine synthase activities by monitoring 5-methyltetrahydrofolate and tetrahydrofolate using high-performance liquid chromatography with fluorescence detection. Anal Biochem 299:253–259. doi:10.1006/abio.2001.5421. 11730351

[68] Wang R, Yi J, Shang J, Yu W, Li Z, Huang H, Xie H, Wang S. 2019. 6-Hydroxypseudooxynicotine dehydrogenase delivers electrons to electron transfer flavoprotein during nicotine degradation by *Agrobacterium tumefaciens* S33. Appl Environ Microbiol 85:e00454-19. doi:10.1128/AEM.00454-19. 30926728 PMC6532033

[69] Pierik AJ, Wolbert RB, Mutsaers PH, Hagen WR, Veeger C. 1992. Purification and biochemical characterization of a putative [6Fe-6S] prismane-cluster-containing protein from *Desulfovibrio vulgaris* (Hildenborough). Eur J Biochem 206:697–704. doi:10.1111/j.1432-1033.1992.tb16976.x. 1318832

