# Supplementary material for: A Heterodimeric Reduced-Ferredoxin-Dependent Methylenetetrahydrofolate Reductase from Syngas-Fermenting Clostridium ljungdahlii
Source: Microbiol Spectr. 2021 Oct 13;9(2):e00958-21. doi: 10.1128/Spectrum.00958-21 (PMC8515935; doi:10.1128/Spectrum.00958-21)
Supplement: SUPPLEMENTAL FILE 1 — Supplemental material. Download SPECTRUM00958-21_Supp_1_seq7.pdf, PDF file, 1.2 MB [file spectrum00958-21_supp_1_seq7.pdf]

Supplemental materials

A heterodimeric reduced-ferredoxin-dependent methylenetetrahydrofolate reductase from syngas-fermenting *Clostridium ljungdahlii*

Jihong Yi,[a] Haiyan Huang,[b] Jiyu Liang,[a] Rufei Wang,[a] Ziyong Liu,[c] Fuli Li,[c] Shuning Wang[a#]

State Key Laboratory of Microbial Technology, Microbial Technology Institute, Shandong University, Qingdao 266237, People's Republic of China[a]; School of Basic Medicine, Shandong First Medical University & Shandong Academy of Medical Sciences, Jinan 250117, People's Republic of China[b]; Qingdao Institute of Bioenergy and Bioprocess Technology, Chinese Academy of Sciences, Qingdao 266101, People's Republic of China[c]

Running head: Fd_{red}^{2-}-dependent methylenetetrahydrofolate reductase

[#]Address correspondence to Shuning Wang, shuningwang@sdu.edu.cn.

Accession	Description	Mass	Score	Matches	Sequences	emPAI	Coverage	
gi|3004370	methylenetetrahydrofolate reductase [Clostridium ljungdahlii DSM 13528]	31756	5802	236(194)	20(19)	96.38	56%	MetF
gi|3004370	conserved hypothetical protein [Clostridium ljungdahlii DSM 13528]	24272	4783	63(50)	16(15)	31.45	74%	MetV
gi|3004371	dihydrolipoamide dehydrogenase [Clostridium ljungdahlii DSM 13528]	49239	809	20(19)	12(12)	3.36	51%	DLDH
gi|3004346	glyceraldehyde-3-phosphate dehydrogenase [Clostridium ljungdahlii DSM 13528]	38836	588	21(18)	13(13)	3.36	43%	
gi|3004366	histidyl-tRNA synthetase [Clostridium ljungdahlii DSM 13528]	48220	431	22(15)	16(13)	1.70	39%	
gi|3004368	predicted aminotransferase [Clostridium ljungdahlii DSM 13528]	40716	399	15(13)	12(12)	5.70	61%	
gi|3004356	predicted zinc-containing alcohol dehydrogenase superfamily [Clostridium ljungdahl	39106	325	15(12)	10(8)	1.65	28%	
gi|3004336	transketolase, subunit B [Clostridium ljungdahlii DSM 13528]	33623	290	10(9)	9(8)	1.33	42%	
gi|3004370	prolyl-tRNA synthetase [Clostridium ljungdahlii DSM 13528]	55114	284	12(10)	9(9)	0.79	21%	
gi|3004336	transketolase, subunit A [Clostridium ljungdahlii DSM 13528]	30173	177	6(5)	5(4)	0.69	20%	
gi|3004334	predicted aminotransferase [Clostridium ljungdahlii DSM 13528]	40716	105	4(4)	4(4)	0.37	5%	
gi|3004345	predicted cell division protein [Clostridium ljungdahlii DSM 13528]	33466	95	5(3)	5(3)	0.46	22%	
gi|3004354	predicted cobalamin B12-binding protein [Clostridium ljungdahlii DSM 13528]	24145	81	1(1)	1(1)	0.14	6%	
gi|3004371	triosephosphate isomerase [Clostridium ljungdahlii DSM 13528]	27440	73	5(3)	4(3)	0.41	18%	
gi|3004353	predicted endonuclease IV [Clostridium ljungdahlii DSM 13528]	33156	70	2(2)	2(2)	0.21	7%	
gi|3004338	predicted cell wall binding protein [Clostridium ljungdahlii DSM 13528]	63844	67	3(2)	3(2)	0.11	4%	
gi|3004374	3-oxoacyl-[acyl-carrier-protein] synthase [Clostridium ljungdahlii DSM 13528]	44499	49	1(1)	1(1)	0.07	3%	
gi|3004338	predicted phosphoesterase [Clostridium ljungdahlii DSM 13528]	26348	44	1(1)	1(1)	0.13	5%	
gi|3004334	predicted hydroxyacid dehydrogenase/reductase [Clostridium ljungdahlii DSM 1352	31796	44	1(1)	1(1)	0.10	4%	
gi|3004367	dihydrodipicolinate reductase [Clostridium ljungdahlii DSM 13528]	27880	43	2(1)	2(1)	0.12	8%	
gi|3004338	predicted capsular polysaccharide biosynthesis protein [Clostridium ljungdahlii DSN	29247	43	2(2)	1(1)	0.24	3%	
gi|3004346	CDP-diacylglycerol-glycerol-3-phosphate 3-phosphatidyltransferase [Clostridium lju	21629	33	2(1)	1(1)	0.16	3%	
gi|3004356	DNA topoisomerase I [Clostridium ljungdahlii DSM 13528]	83932	26	1(1)	1(1)	0.04	0%	
gi|3004335	cyanophycin synthetase [Clostridium ljungdahlii DSM 13528]	97425	17	3(0)	1(0)	0.03	0%	
gi|1025697	hypothetical protein WY13_02572 [Clostridium ljungdahlii]	55105	15	1(1)	1(1)	0.06	1%	

FIG S1 The peptide mass fingerprinting analysis of purified MTHFR from *C. ljungdahlii*. The top three identified proteins are MetF, MetV and DLDH, respectively.

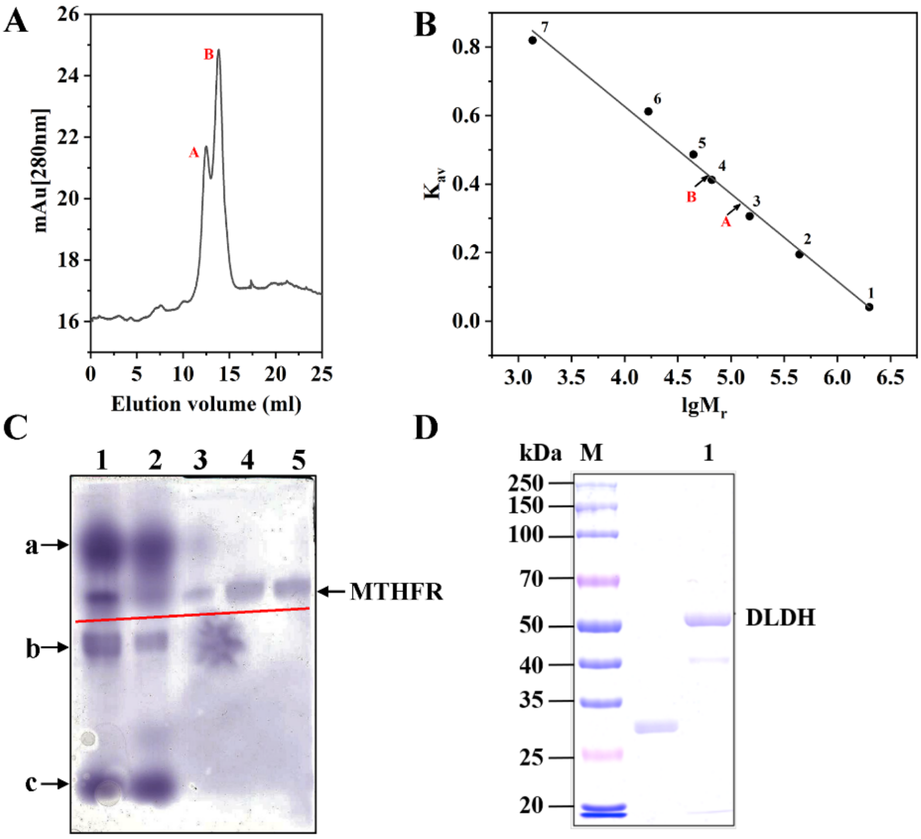

FIG S2 (A) and (B) Determination of relative molecular mass of the purified MTHFR from *C. ljungdahlii* on a GE Superdex G200 column (10 by 300 mm). The size standards used: 1. dextran blue, 2,000 kDa; 2. ferritin, 440 kDa; 3. γ-globin, 158 kDa;

4. BSA, 66 kDa; 5. chicken serum protein, 44.3 kDa; 6. ferroheme, 16.7 kDa 7. vitamin B12, 1,360 Da. According to the standard curve, peak A is corresponding to the tetrameric form of the enzyme with an apparent molecular mass as 132.3 kDa, and peak B is corresponding to the heterodimeric form of the enzyme with an apparent molecular mass as 62.2 kDa. (C) Native–PAGE and activity staining (reduction of BV with methyl-THF) analysis of the purification of MTHFR from *C. ljungdahlii*. The experiment was performed in the anaerobic chamber filled with 95% N_2–5% H_2. The activity staining solution contained 50 mM potassium phosphate buffer (pH7.3), 10 mM BV, and 0.5 mM methyl-THF. A. Lane 1, cell extracts; lane 2, pooled fractions from ammonium sulfate precipitation; lane 3, pooled fractions from Phenyl-Sepharose; lane 4, pooled fractions from Q-Sepharose; lane 5, pooled fractions from DEAE-Sepharose. There are three non-specific bands (a, b, and c) in lanes 1 and 2, which should be other dehydrogenases/reductases because there is only one set of genes for MTHFR in the genome of *C. ljungdahlii*. Since H_2 in the anaerobic chamber can also reduce BV, one of them was a hydrogenase. Considering that methylene-THF was produced in the MTHFR-catalyzed activity staining reaction, there was probably a methylene-THF dehydrogenase. There might also be a glycine dehydrogenase (a component of glycine cleavage system) since glycine was used in the electrophoresis buffer. The three enzymes were removed in subsequent purification steps. (D) SDS–PAGE analysis of the purified DLDH of *C. ljungdahlii*. The enzyme was separated from MTHFR by Q-Sepharose during the purification of MTHFR. M, protein marker; lane 1, the purified DLDH.

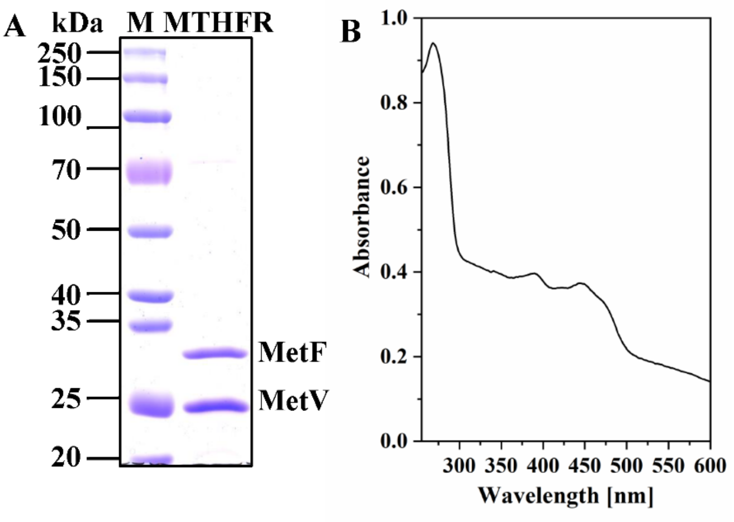

FIG S3 SDS-PAGE analysis (A) and UV-visible absorption spectrum (B) of purified recombinant MTHFR of *C. ljungdahlii*.

Table S1 Enzyme assay of the purified MTHFR from *C. ljungdahlii* using NAD(P)H as the electron donor.

**Substrates**	**Specific enzyme activity (U/mg)**
**NADH**[a] + methylene-THF	ND[b]
**NADPH** + methylene-THF	ND
**NADPH** + NAD^+ + methylene-THF	ND
**NADH** + $NADP^+$ + methylene-THF	ND
NADPH + **Fd** + methylene-THF	ND
NADH + **Fd** + methylene-THF	ND

[a]Boldfacing indicates substrates or products whose reduction or oxidation were monitored spectrophotometrically;
[b]ND, not detectable

Table S2 Enzyme assay of the purified MTHFR from *C. ljungdahlii* by coupling with DLDH-catalyzed reaction.

**Substrates**	**Specific enzyme activity (U/mg)**
NADH + lipoamide + methylene-THF[a]	ND[b]
NADPH + lipoamide + methylene-THF[a]	ND
NADH + methylene-THF	ND
NADPH + methylene-THF	ND
NADH + Fd + methylene-THF	ND
NADPH + Fd + methylene-THF	ND
NADH + $NADP^+$ + methylene-THF	ND
NADPH + NAD^+ + methylene-THF	ND

[a] The reaction was monitored by the formation of methyl-THF using HPLC.
[b]ND, not detectable

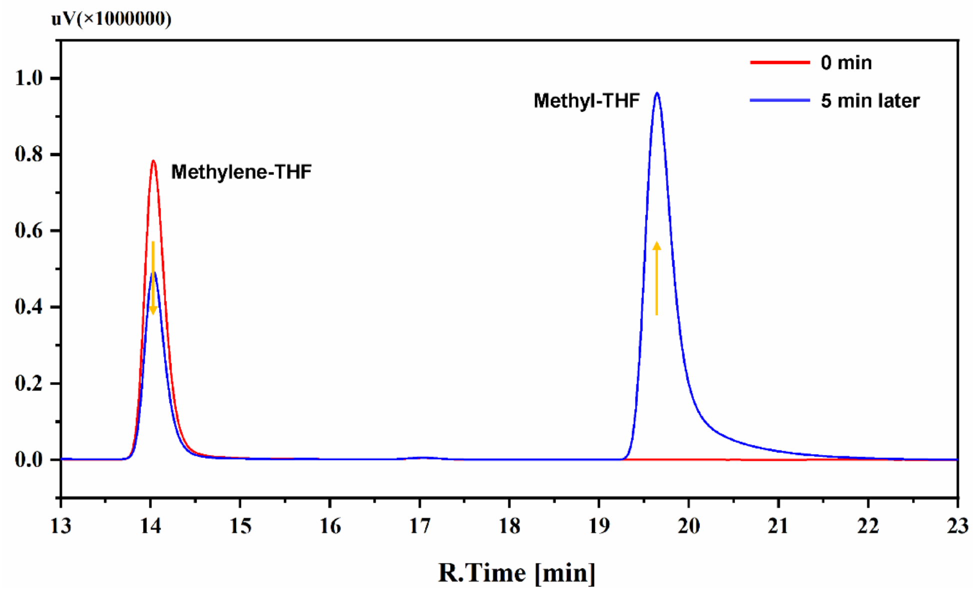

FIG S4 HPLC profiles for monitoring the formation of methyl-THF. The red line was the sample at the beginning of the reaction, and the blue line was the sample taken 5 min later. The response value of methyl-THF is larger than that of methylene-THF with the fluorescence detection method.

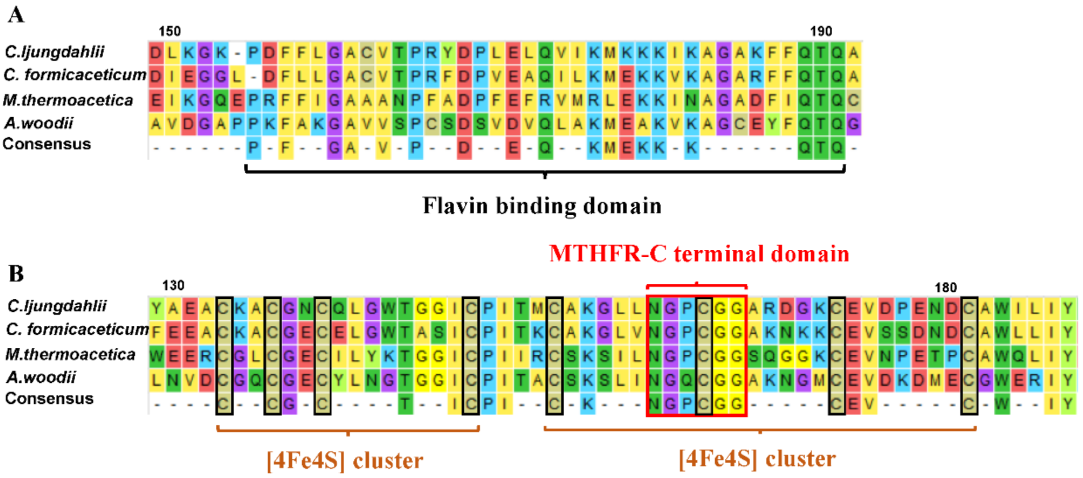

FIG S5 Partial protein sequence alignment and conserved domain analysis of MetF (A) and MetV (B) from *C. ljungdahlii* (CLJU_RS18520-25), *C. formicaceticum* (CLFO_11300-290), *M. thermoacetica* (MOTHE_c116-80), and *A. woodii* (Awo_c09310-00). MetF subunit contains a conserved flavin binding domain, and MetV subunit contains two predicted [4Fe4S] clusters and a MTHFR-C terminal domain, where a motif NGPCGG is conserved.

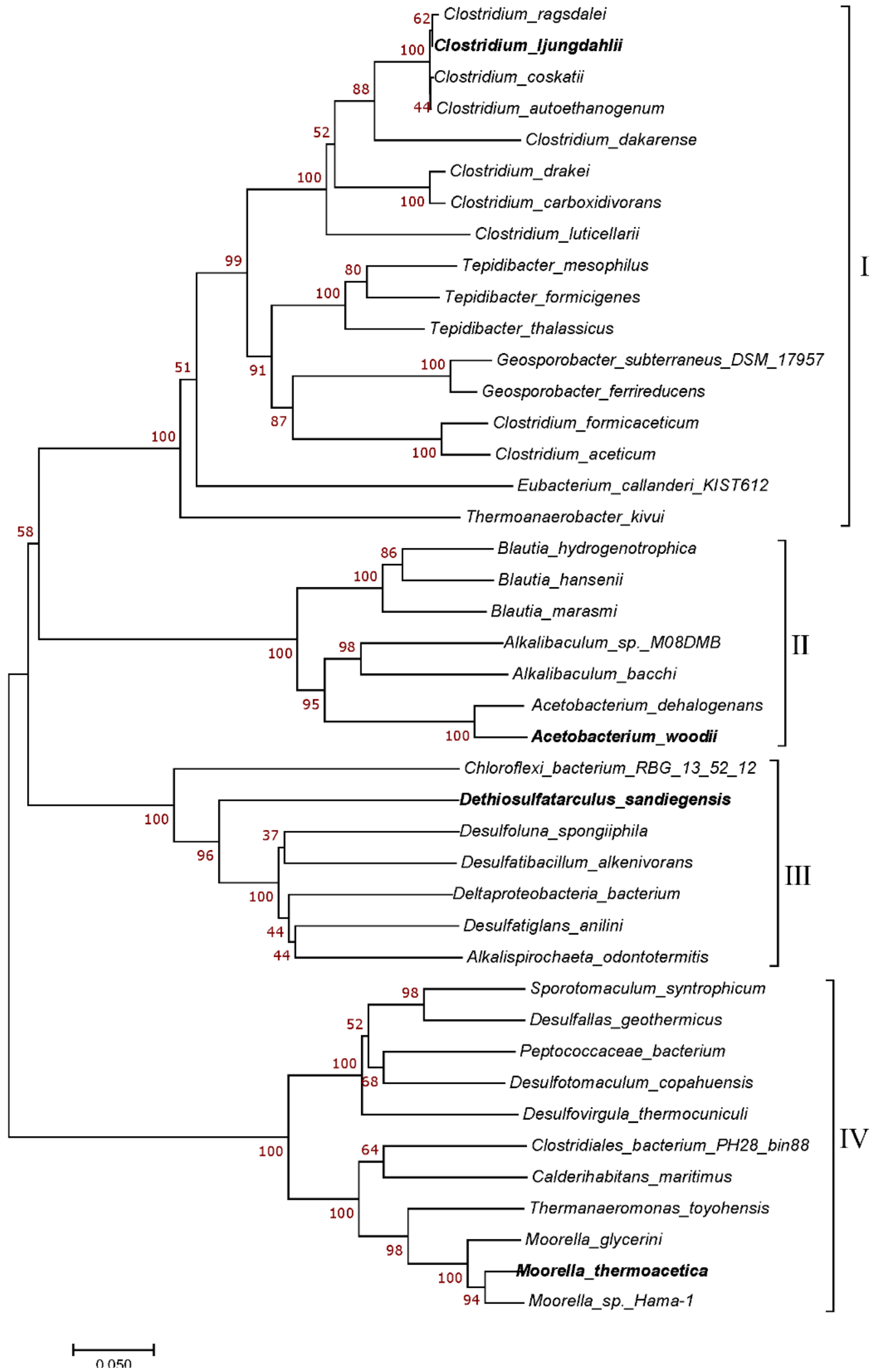

FIG S6 Phylogenetic analysis of the MetFV-type MTHFR from different bacteria. We totally found more than 100 MetFV homologs in GenBank with protein sequence identity higher than 35% to *C. ljungdahlii* MetFV, all of which are from anaerobes. The phylogenetic tree showed three groups of MetFV, Group I is corresponding to the dimeric MTHFR represented by *C. ljungdahlii* MetFV, Group II is corresponding to trimeric MTHFR represented by *A. woodii* MetFV-RnfC2 complex, Group III is

corresponding to hypothetical Pentamer MTHFR represented by *D. sandiegensis* MetFV-NuoEFG complex, and Group IV is corresponding to hexameric MTHFR represented by *M. thermoacetica* MetFV-MvhD-HdrABC complex.

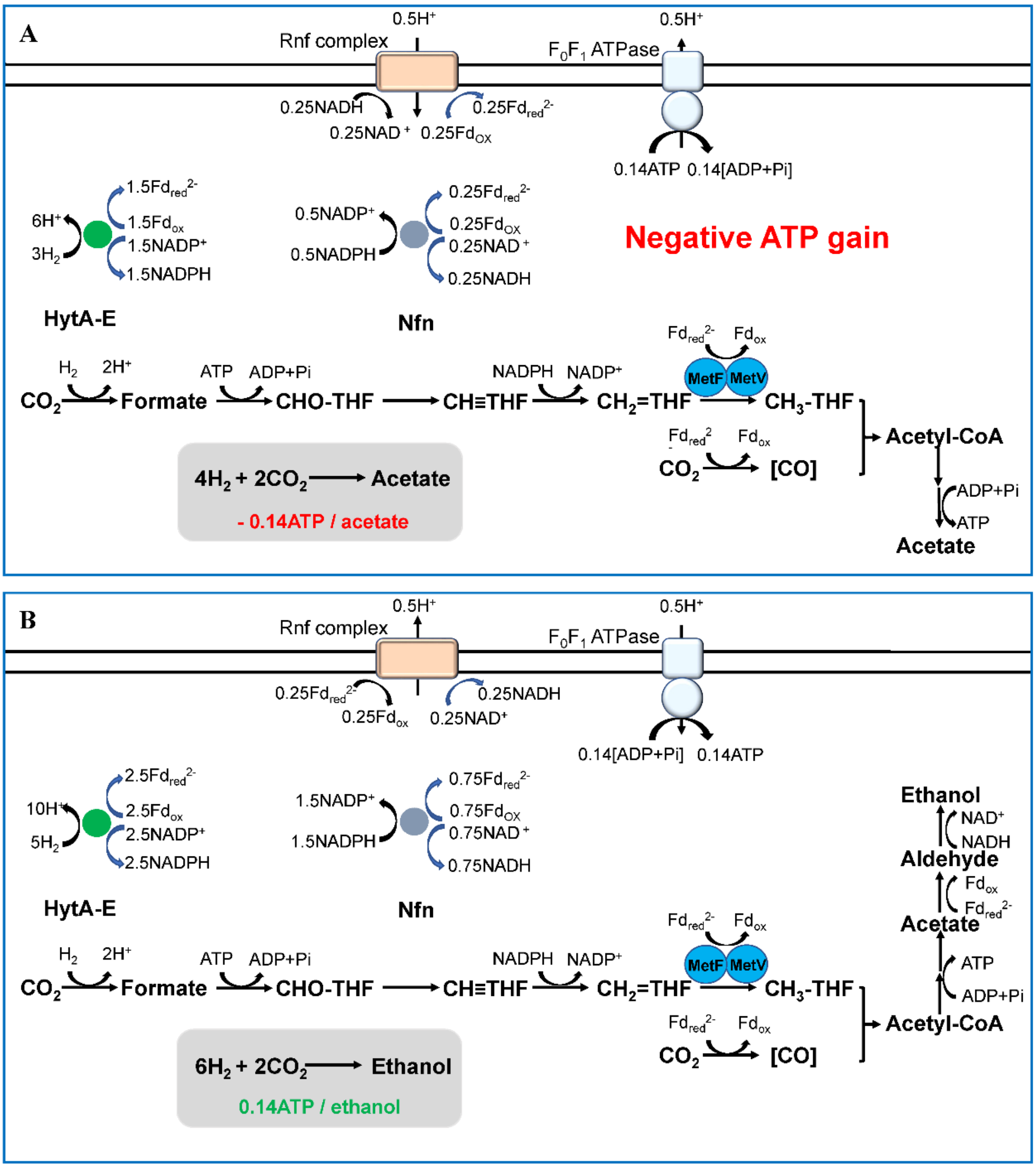

FIG S7 Schemes of the energy metabolism of *C. ljungdahlii* grown on H_2 and CO_2, assuming that only acetate (A) or ethanol (B) is formed, and that the Rnf-F_1F_0ATPase system can drive the reduction of Fd with NADH by hydrolyzing ATP.
